# LPS-induced TNF-α factor mediates pro-inflammatory and pro-fibrogenic pattern in non-alcoholic fatty liver disease

**DOI:** 10.18632/oncotarget.5163

**Published:** 2015-10-08

**Authors:** Sara Ceccarelli, Nadia Panera, Marco Mina, Daniela Gnani, Cristiano De Stefanis, Annalisa Crudele, Chiara Rychlicki, Stefania Petrini, Giovannella Bruscalupi, Laura Agostinelli, Laura Stronati, Salvatore Cucchiara, Giovanni Musso, Cesare Furlanello, Gianluca Svegliati-Baroni, Valerio Nobili, Anna Alisi

**Affiliations:** ^1^ Liver Research Unit, “Bambino Gesù” Children's Hospital-IRCCS, Rome, Italy; ^2^ Hepato-Metabolic Disease Unit, “Bambino Gesù” Children's Hospital-IRCCS, Rome, Italy; ^3^ Predictive Models for Biomedicine and Environment Unit, Fondazione Bruno Kessler, Trento, Italy; ^4^ Department of Gastroenterology, Polytechnic University of Marche, Ancona, Italy; ^5^ Confocal Microscopy Core Facility, “Bambino Gesù” Children's Hospital-IRCCS, Rome, Italy; ^6^ Department of Biology and Biotechnology “C. Darwin”, Sapienza University of Rome, Rome, Italy; ^7^ Department of Radiobiology and Human Health, ENEA, Rome, Italy; ^8^ Pediatric Gastroenterology and Liver Unit, Sapienza University of Rome, Rome, Italy; ^9^ Gradenigo Hospital, Turin, Italy; ^10^ Center for Obesity, Polytechnic University of Marche, Ancona, Italy

**Keywords:** Pathology Section, fibrosis, LITAF, LPS, inflammation, NAFLD

## Abstract

Lipopolysaccharide (LPS) is currently considered one of the major players in non-alcoholic fatty liver disease (NAFLD) pathogenesis and progression. Here, we aim to investigate the possible role of LPS-induced TNF-α factor (LITAF) in inducing a pro-inflammatory and pro-fibrogenic phenotype of non-alcoholic steatohepatitis (NASH).

We found that children with NAFLD displayed, in different liver-resident cells, an increased expression of LITAF which correlated with histological traits of hepatic inflammation and fibrosis. Total and nuclear LITAF expression increased in mouse and human hepatic stellate cells (HSCs). Moreover, LPS induced LITAF-dependent transcription of IL-1β, IL-6 and TNF-α in the clonal myofibroblastic HSC LX-2 cell line, and this effect was hampered by LITAF silencing. We showed, for the first time in HSCs, that LITAF recruitment to these cytokine promoters is LPS dependent. However, preventing LITAF nuclear translocation by p38MAPK inhibitor, the expression of IL-6 and TNF-α was significantly reduced with the aid of p65NF-ĸB, while IL-1β transcription exclusively required LITAF expression/activity. Finally, IL-1β levels in plasma mirrored those in the liver and correlated with LPS levels and LITAF-positive HSCs in children with NASH.

In conclusion, a more severe histological profile in paediatric NAFLD is associated with LITAF over-expression in HSCs, which in turn correlates with hepatic and circulating IL-1β levels outlining a panel of potential biomarkers of NASH-related liver damage. The *in vitro* study highlights the role of LITAF as a key regulator of the LPS-induced pro-inflammatory pattern in HSCs and suggests p38MAPK inhibitors as a possible therapeutic approach against hepatic inflammation in NASH.

## BACKGROUND

Over the past few years, the study of the gut-liver axis involvement in the pathogenesis of chronic liver diseases has become of central importance. Notably, it has been reported that the Toll-like receptor (TLR)-4 signalling cascade initiated by gut endotoxins, including lipopolysaccharide (LPS), is involved in the development and progression of chronic liver injury [1]. Importantly, several lines of evidence have described the involvement of the LPS/TLR-4 pathway in non-alcoholic fatty liver disease (NAFLD) pathogenesis and in its progression to the more severe form of non-alcoholic steatohepatitis (NASH) [1, 2]. A close relationship between augmented circulating LPS levels, fibrosis and NASH severity has been reported both in human subjects and in animal models. In particular, clinical studies have shown increased LPS plasma levels in the systemic and portal circulation of patients with cirrhosis [3, 4]. Accordingly, obese paediatric patients with NAFLD/NASH display elevated serum LPS levels, a finding which correlates with the severity of disease [5]. Moreover, in NAFLD/NASH animal models the disruption of intestinal integrity leads to increased LPS translocation, TLR-4 signalling activation, hepatic inflammation and fibrogenesis [6]. Coherently, the blockage of LPS/TLR-4 signalling, via genetic ablation of *TLR-4* or via alteration of intestinal microbiota either by antibiotics or by probiotics protects patients from diet-induced NAFLD and fibrosis [7, 8]. Furthermore, an important role for gut microbiota imbalance has been suggested in NASH patients, who exhibited a sterile pro-inflammatory pattern and an augmented hepatic TLR-4 expression [9, 10]. Studies also have shown that TLR-4/dysbiosis plays a critical role in the progression of NAFLD [11, 12].

The LPS-induced tumour necrosis factor (TNF)-α factor (LITAF), alternatively known as small integral membrane protein of the lysosome/late endosome (SIMPLE) and as p53 inducible gene-7 (PIG-7) protein, has been initially identified as a p53-inducible target in DLD-1 colon cancer cell lines [13]. Together with nuclear factor kappa-B (NF-ĸB), LITAF has been identified as a novel cis-acting regulatory protein crucial for human LPS-dependent transcription of *TNF*-α gene in human monocytic THP-1 leukaemia cells [14]. The *LITAF* gene maps to chromosome 16p12–16p13.3 in humans and high levels of its mRNA are found mainly in placenta, peripheral blood leukocytes, lymph nodes and spleen [14]. The LITAF protein is primarily expressed in monocytes/macrophages and spleen, but also in bone marrow, brain, heart, lung and liver [15]. Importantly, whole-body *LITAF* deficiency has a dramatic effect on systemic and chronic local inflammatory responses [15]. LITAF is currently considered one of the most important players in the activation of pro-inflammatory molecules under LPS stimulation in macrophages [16, 17]. Specifically, Tang et al. demonstrated, through footprinting analysis, that the human LITAF binds a CTCCC (−515 to −511) responsive element within the *TNF*-α promoter [16]. The authors further identified a *LITAF* region (amino acids 165–180) that mediates the binding between LITAF and the *TNF*-α promoter, called the peptide B [16]. They also demonstrated that macrophage-specific LITAF-deficient mice (mac*LITAF*−/−) exhibited a resistance to LPS-induced lethality and a decreased expression of various cytokines, including TNF-α, interleukin (IL)-6, soluble TNF-α receptor II and chemokine CXCL16 [17].

LITAF has been indicated as an important player connecting inflammation, obesity and associated disorders, such as NAFLD. In particular, a positive correlation between LITAF expression in human peripheral monocytes and obesity, insulin resistance and plasmatic pro-inflammatory cytokines levels has been reported [18]. We recently demonstrated that LITAF mRNA and protein expression is up-regulated in rat models with diet-induced NAFLD. In light of this, we proposed that this molecule could be a novel factor for mediating the LPS/TLR-4 axis during development and progression of the disease [19]. Despite plentiful evidence regarding LITAF regulation in macrophage populations, to date no studies have clarified LITAF involvement in the pro-inflammatory and pro-fibrogenic pathways related to liver disorders. Since increased endotoxemia and TLR-4 signalling play a major role in the activation of pro-inflammatory and pro-fibrogenic phenotype in hepatic stellate cells (HSCs), the potential role of LITAF in these mechanisms should be investigated [7].

Here, we show that LITAF expression and activity increase under LPS exposure. We identify LITAF as a key regulator of the LPS-induced pro-inflammatory pattern in HSCs. Accordingly, the increased expression of LITAF in whole liver and in HSCs is strongly associated with a more severe histological profile of inflammation and fibrosis in children with NAFLD. Moreover, our data suggests the use of p38MAPK inhibitors as a promising therapeutic approach to treating hepatic inflammation in NASH.

## RESULTS

### Children with NAFLD exhibit hepatic over-expression of LITAF as a tracer of liver damage progression

We previously reported an up-regulation of *LITAF* transcript consistent with the increased expression of hepatic LITAF protein levels in high-fat/high-fructose diet-induced NAFLD in rats [19]. In the present study, we analysed expression levels of *LITAF* mRNA and protein in children with biopsy-proven NAFLD. The diagnosis of NAFLD was established following a standard clinical and histological workup as previously described [20]. Sample collection and use was performed after obtaining approval of the Ethical Committee of the Bambino Gesù Children's Hospital and written consent by parents of the children.

**Figure 1:**
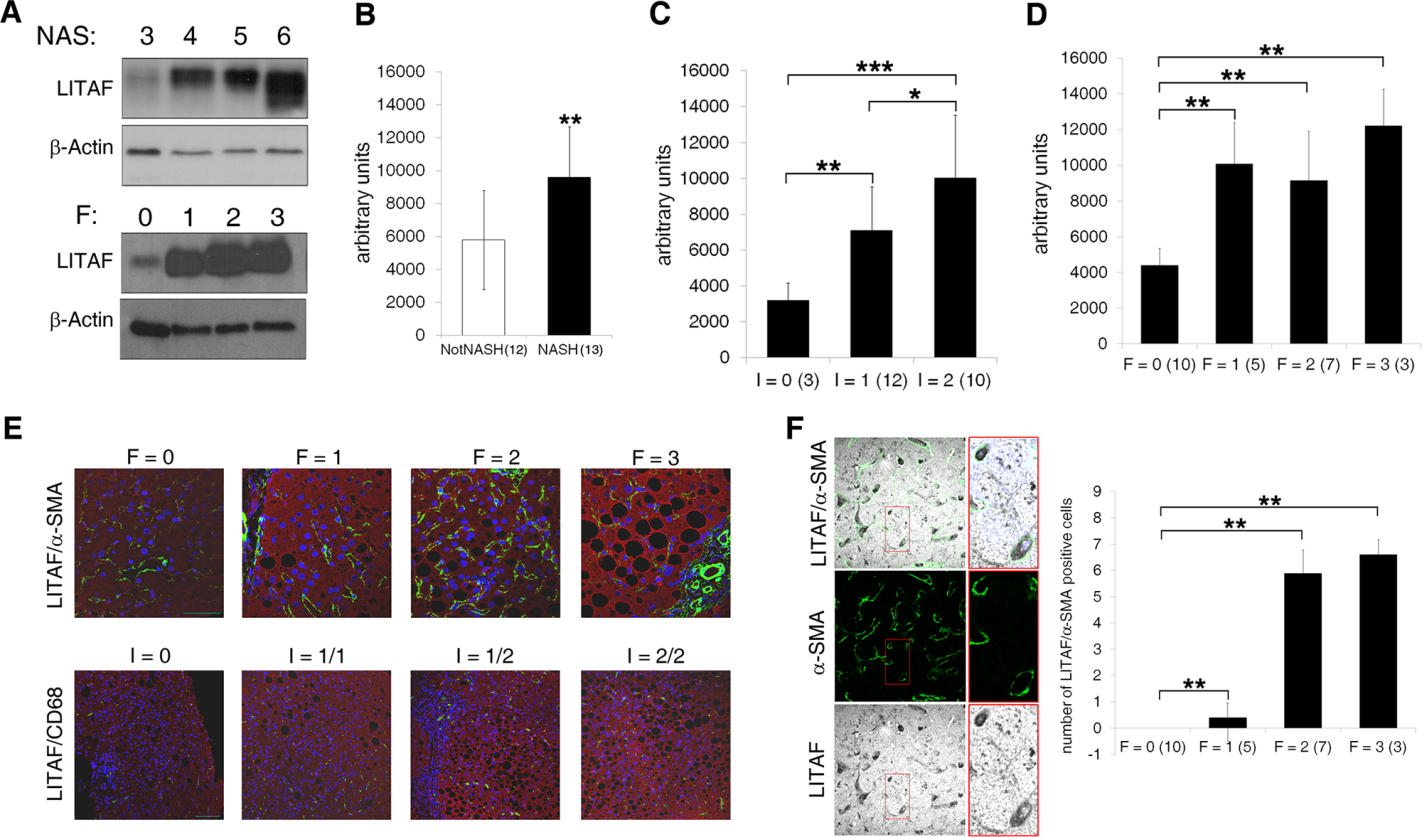
Hepatic LITAF expression increases in NAFLD children correlating with histological traits of hepatic inflammation and fibrosis **A.** Immunoblot analysis of total LITAF protein expression in liver from NAFLD children according to NAS and fibrosis (*n* = 25). The immunoblot is representative of 3 different Western Blottings. Lanes were run on the same gel but were non-contiguous. **B–D.** Quantitative densitometric analysis of LITAF protein expression in patients (B) with NASH *vs*. NotNASH, (C) with I = 0 *vs*. I = 1 *vs*. I = 2 and (D) F = 0 *vs*. F = 1, *vs*. F = 2 *vs*. F = 3. Histogram represents the mean values ± standard deviation (SD). **E.** Representative confocal imaging of α-SMA/LITAF (green/red) and CD68/LITAF (green/red) respect to fibrosis (0–3) and inflammation (0–2) grading in liver tissues from NAFLD children (*n* = 25). The nuclei are in blue (scale bar: 50 μm for fibrosis; scale bar: 100 μm for inflammation). **F.** Representative confocal laser microscopy of LITAF (grey) and α-SMA (green) in liver tissues from NAFLD children. The two labels are merged with nuclei. Nuclei were counterstained with DAPI (blue) (scale bar: 50 μm). The histogram represents the mean ± SD of LITAF/α-SMA positive HSCs in samples with F = 0 *vs*. F = 1 *vs*. F = 2 *vs*. F = 3 (*n* = 25). Differences across groups were analysed by Student's two-tailed or ANOVA as appropriate. *p < 0.05, **p < 0.01, ***p < 0.001.

The analysis of the liver protein expression showed a significant increase of LITAF levels corresponding to disease severity progression measured in terms of NAFLD activity score (NAS), and the presence of NASH (Fig. 1A, upper panels and 1B). Moreover, LITAF protein expression levels increased consistently with the severity of fibrosis (Fig. 1A, lower panels and 1D) and of inflammation (Fig. 1C) assessed by Kleiner scores [21]. Quantitative Real Time-PCR (qRT-PCR), revealed not significant changes in mean *LITAF* mRNA levels with respect to the presence of NASH and grading of inflammation and fibrosis (Fig. S1A).

Whatever aetiology, liver fibrosis is a wound healing response to liver injury, involving proliferation and activation of HSCs towards a myofibroblast phenotype that accumulates stress fibers, mainly characterized by α-smooth muscle actin (α-SMA) over-expression [22, 23]. As shown Fig. 1E, the increased severity of fibrosis and inflammation, expressed in terms of both histological scores and α-SMA or CD68 positivity for macrophages, correlated with LITAF up-regulation in children with NAFLD. Interestingly, the number of CD163/LITAF positive M2 macrophages increased with severity of inflammation (Fig. S1B).

Importantly, the number of LITAF/α-SMA positive HSCs was higher in NAFLD children with F = 1 than in those with F = 0, and continued to increase with fibrosis progression (Fig. 1F). Moreover, α-SMA positive HSCs and cytokeratin 8/18 (CK8/18) positive hepatocytes exhibited an increased expression of LITAF protein in NAFLD livers compared to those from 8 healthy children (Fig. S1C).

### Activated primary mouse HSCs display serum-dependent increase of LITAF expression/activity and inflammation

**Figure 2:**
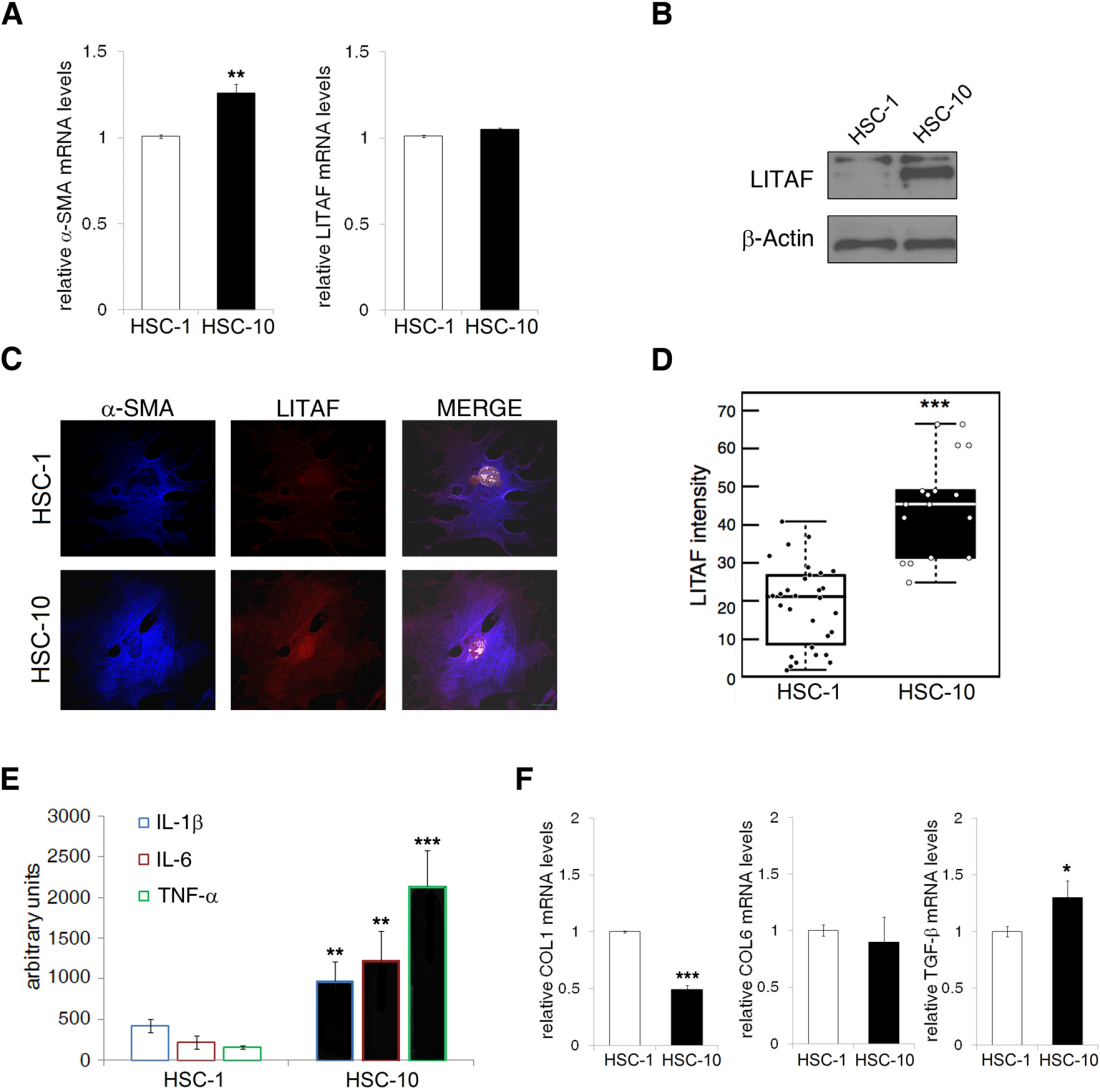
LITAF expression/activity and inflammatory response is upsized by serum in activated primary mouse HSCs **A–F.** Primary mouse activated HSCs were serum starved for 24 h and then treated with either 1% (HSC-1) or 10% FBS (HSC-10) at different timepoints. A: QRT-PCR analyses of α-*SMA* and *LITAF* mRNA expression at 2 h (*n* = 3). B: Immunoblot analysis of total LITAF at 3 h (data presented is representative of three independent experiments). C: Representative confocal imaging of α-SMA (blue), LITAF (red) and nuclei (stained with DRAQ5, white) at 2 h (scale bar: 20 μm). D: Quantitative imaging analysis of LITAF nuclear translocation (n cells > 50). Mann-Whitney *U* test. E: Semi-quantitative protein expressions of IL-1β, IL-6 and TNF-α in total cell lysate of HSC-1 and HSC-10 by using a Cytokine Antibody Array (*n* = 2 in duplicate) at 3 h. F: QRT-PCR analyses of COL1, COL6 and TGF-β at 2 h (*n* = 3). Histograms represent the mean ± SD. *p < 0.05; **p < 0.01; ***p < 0.001 *vs*. HSC-1, Student's *t* test.

Primary cultured mouse HSCs were activated on plastic, serum starved for 24 h, and then treated with either 1% (HSC-1) or 10% FBS (HSC-10) at different timepoints (2, 3 and 6 h). We first explored the expression of *LITAF* mRNA and protein in HSC-1 and HSC-10 at 2 h. We found a significant increase of α-*SMA* mRNA but no change of *LITAF* mRNA levels in HSC-10 compared to HSC-1 (Fig. 2A). However, Western Blotting analysis showed that LITAF protein, almost absent in HSC-1, is strongly up-regulated in HSC-10 at 3 h (Fig. 2B). As Tang et al [17] demonstrated that the transcriptional activity of LITAF could depend on its nuclear translocation in macrophages, we then investigated LITAF and α-SMA intra-cellular distribution. Confocal laser microscopy showed that at 2 h LITAF and α-SMA expression were higher in HSC-10 than HSC-1 (Fig. 2C). Furthermore, quantitative imaging analysis performed by a novel dedicated bioinformatic pipeline (Fig. S2A) revealed that LITAF nuclear amount was predominant in HSC-10 (Fig. 2D).

Once activated, HSCs migrate and accumulate in the damaged sites secreting a large quantity of extracellular matrix proteins and fibrillar collagens [22, 24–27]. The autocrine and paracrine perpetuation of HSC activation is sustained by increased production and/or activity of a wide array of cytokines [25]. Accordingly, by the usage of a sensitive antibody (Ab) array approach containing Abs against 40 specific cytokines (Fig. S2B), we found that the expression of several pro-inflammatory cytokines, including IL-1β, IL-6 and TNF-α, was significantly up-regulated in the HSC-10 with respect to HSC-1 phenotype after 3 h from FBS supplementation (Fig. 2E and Fig. S2C and S2D). It is well known that HSCs can change to a myofibroblast-like activated phenotype, characterized by the increased α-SMA expression and by production and secretion of a large amounts of collagens, including type I (COL1) and type VI (COL6) collagens, and pro-fibrogenic cytokines, such as transforming growth factor (TGF)-β [28]. However, we found that the prolongation of the activation state by 10% FBS had no further activating effects on HSCs. In fact, qRT-PCR confirmed that *COL1* mRNA significantly decreased, *TGF*-β mRNA significantly increased and *COL6* remained unaltered in HSC-10 compared to HSC-1 at 2 h (Fig. 2F). All these results indicate that the presence of 10% FBS medium enhances the pro-inflammatory phenotype and only partially that pro-fibrogenic.

### LPS induces LITAF expression/activity in primary mouse activated HSCs

**Figure 3:**
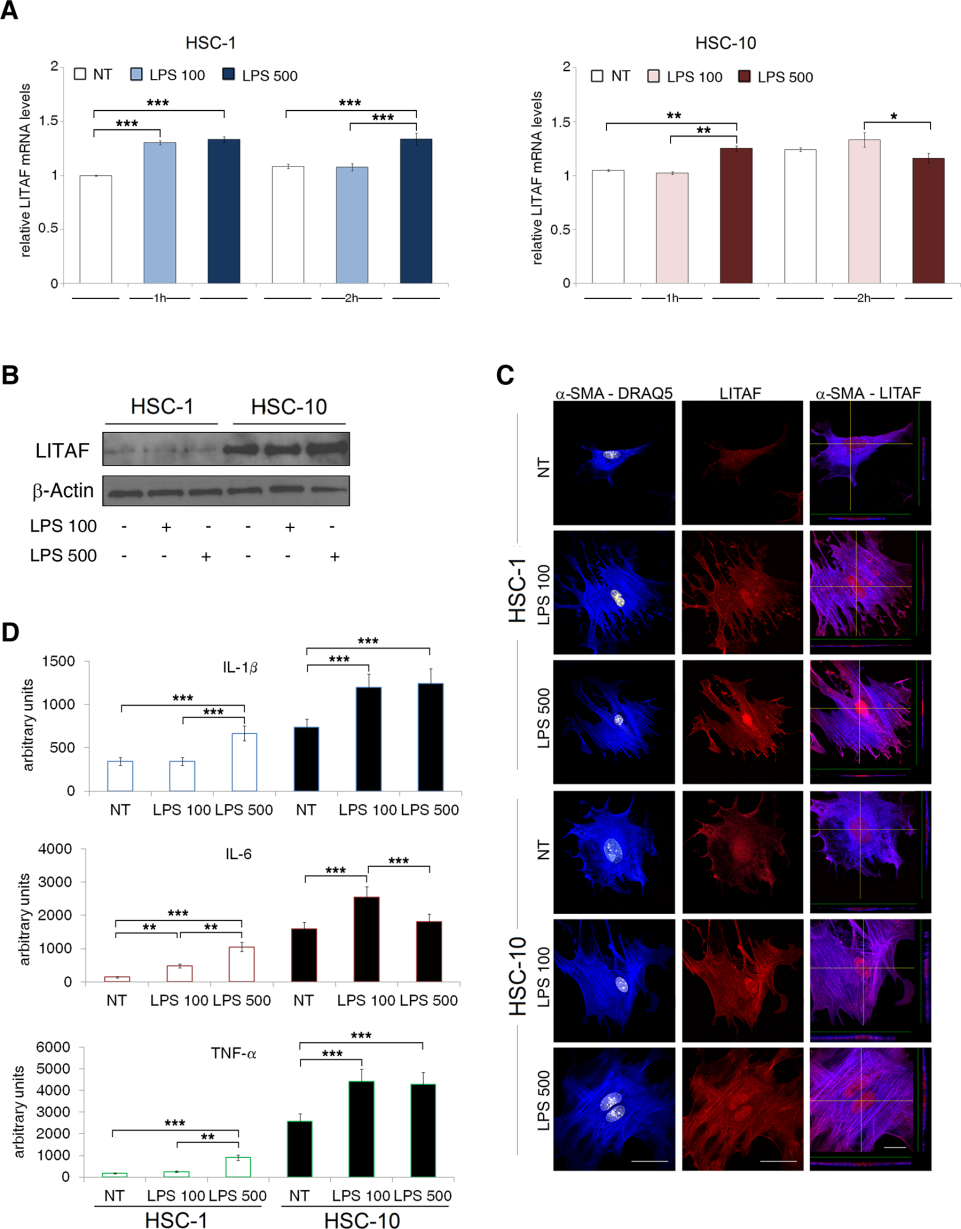
LPS increases LITAF and pro-inflammatory markers expression in HSC-1 and HSC-10 **A–D.** Isolated HSC were cultured for 5 days in medium supplemented with 10% FBS and then serum starved for additionally 24 hours. Cells were then stimulated with LPS (100 and 500 ng/ml) in the presence of either 1% (HSC-1) or 10% (HSC-10) FBS. Cells were then collected at different timepoints. A: QRT-PCR analysis of *LITAF* mRNA expression (*n* = 3). B: Immunoblot analysis of total LITAF at 3 h (data presented is representative of three independent experiments). C: Representative confocal imaging of α-SMA (blue), LITAF (red) and nuclei (white), and X- and Y-axis projections of Z-reconstructions (right column) of α-SMA/LITAF double-stained cells at the level of LITAF nuclear localization after 2 h of LPS treatment. Nuclei were counterstained with DRAQ5 (scale bar: 50 μm). D: Semi-quantitative protein expressions of IL-1β, IL-6 and TNF-α in total cell lysate of HSC-1 and HSC-10 after 3 h by using a Cytokine Antibody Array (*n* = 2). Histograms represent the mean ± SD. Differences across groups were analysed by ANOVA. *p < 0.05; **p < 0.01; ***p < 0.001.

The liver exposure to gut-derived microbial products, such as LPS, and the resulting activation of TLR-4 signalling in HSCs is now emerging as one of the key event in NAFLD-related fibrosis and inflammation [29, 30]. In order to investigate whether endotoxin may exacerbate LITAF expression and/or nuclear translocation we treated primary mouse HSC-1 and HSC-10 with two different concentrations of LPS (100 or 500 ng/ml) at different timepoints (1, 2 and 3 h). Our results demonstrated that the highest concentration of LPS (500 ng/ml) significantly increased *LITAF* mRNA levels (Fig. 3A) both in HSC-1 and HSC-10 early after 1 h of treatment, accordingly to an up-regulation of LITAF protein expression observed at 3 h (Fig. 3B). However, confocal laser microscopy (Fig 3C) highlighted that both LPS concentrations induced a significant increase of LITAF nuclear translocation, accompanied by a concomitant rise in α-SMA expression after 2 h from treatment either in HSC-1 or in HSC-10. These results were confirmed by quantitative imaging data and highlighted that nuclear translocation of LITAF increased mainly in HSC-10 as early as 1 h after LPS addition (Fig. S3A).

In macrophages, LPS is the main factor activating LITAF transcriptional activity of different cytokines, particularly TNF-α [17]. Therefore, we next analysed the LPS effect on autocrine production of a wide array of cytokines. The array profile showed LPS-induced production of several cytokines (Fig. S3B). Particularly, after 3 h treatment, LPS caused a significant up-regulation of IL-1β, IL-6 and TNF-α production with respect to untreated cells in both HSC-1 and HSC-10 (Fig. 3D). Furthermore, we found that LPS treatment caused an increase of *α-SMA* and *TGF-β* gene expression (Fig. S3C and S3D). Whereas other pro-fibrogenic genes, such as *COL1* and *COL6*, were fluctuating depending on LPS dose and time exposure (Fig. S3E and S3F).

### LPS-dependent pro-inflammatory phenotype in human HSCs is mediated by LITAF transcriptional activity

**Figure 4:**
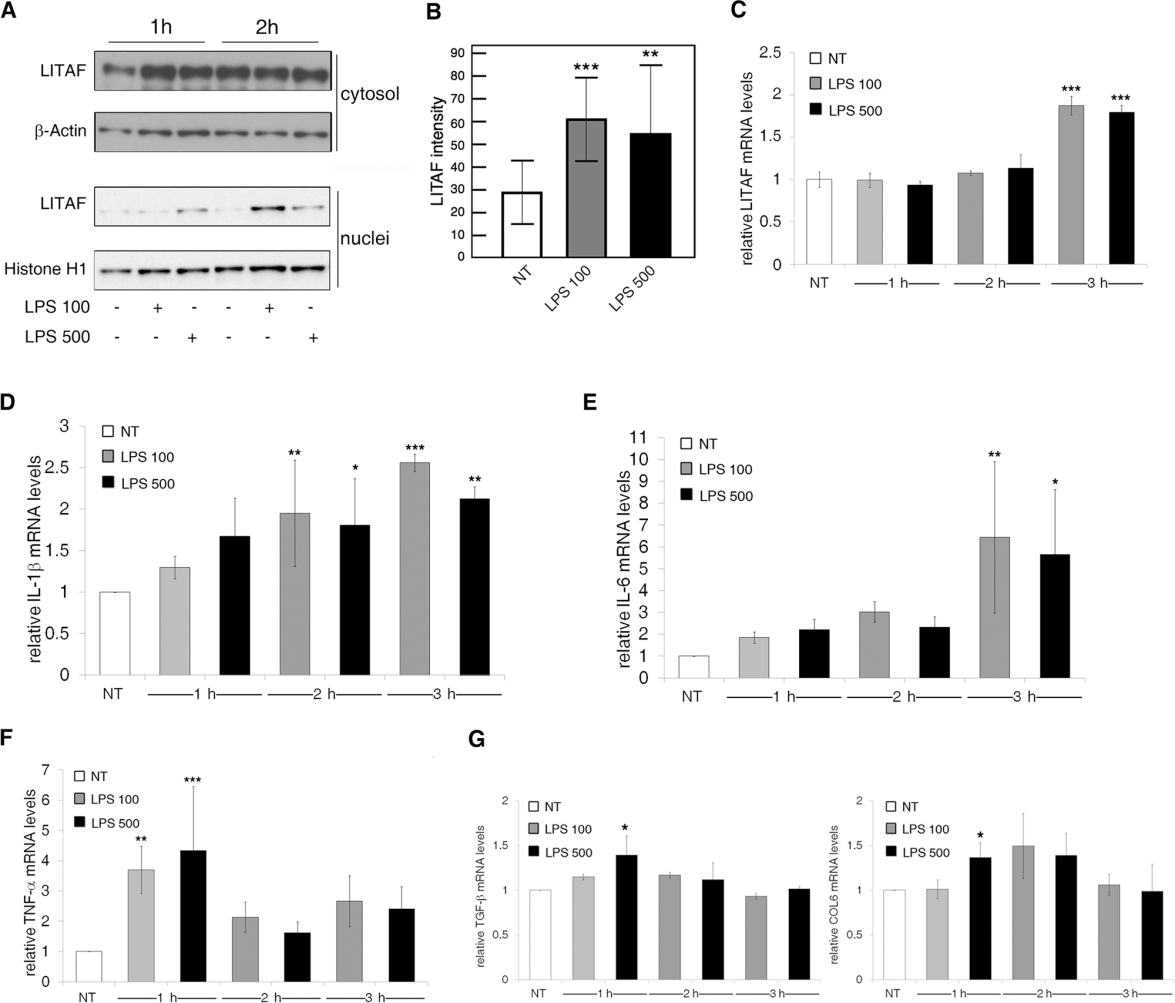
LPS induces LITAF nuclear translocation and inflammatory and fibrotic responses in LX-2 cells **A–G.** LX-2 cells with (LPS 100 and LPS 500) or without (NT) LPS treatment. A: Immunoblot analysis of cytosolic and nuclear LITAF after 1 h and 2 h from LPS treatment (data presented is representative of three independent experiments). B: Quantitative imaging analysis of LITAF nuclear translocation after 2 h from LPS addition. Mann-Whitney *U* test. C-G: QRT-PCR analyses of (C) *LITAF*, (D) *IL-1β*, (E) *IL-6*, (F) *TNF-α* and (G) *TGF-β* and *COL6* mRNA after 1 h, 2 h and 3 h from LPS stimulus (*n* = 3). Histograms represent the mean ± SD. Differences across groups were analysed by ANOVA. *p < 0.05; **p < 0.01; ***p < 0.001 *vs*. NT.

In light of the previously reported evidence, we examined the LPS-dependent LITAF expression and distribution in LX-2 HSC cell line. This cell line, that has been extensively validated for its similarities to human culture activated HSCs, presents a higher efficiency of transfectability and expression of ectopic genes and silencing than primary HSCs [31]. In detail, we stimulated LX-2 cells with different dosages of LPS (100 or 500 ng/ml) at different timepoints (1, 2, 3 and 24 h). Coherently with the results obtained in primary mouse HSCs, LITAF protein was more abundant into the nucleus of LX-2 cells early after 1 h and peaked at 2 h from LPS stimulation with respect to the untreated counterpart (Fig. 4A and Fig. S4A) as also confirmed by a quantitative analysis of imaging data (Fig. 4B). As observed in primary mouse HSCs, total *LITAF* mRNA significantly increased after 3 h from LPS stimulus, either at 100 or 500 ng/ml (Fig. 4C). Interestingly, we also reported that LPS inhibited LX-2 cell proliferation measured as BrdU incorporation at 24 h (Fig. S4B). Considering the LPS-dependent pro-inflammatory and pro-fibrogenic phenotype observed in primary mouse HSCs, we further analyzed in LX-2 a panel of cytokines and various well-established molecular markers of transdifferentiation. In order to delineate the modulation of each target gene along the time we exposed LX-2 cells to LPS within a time window from 1 h to 3 h. We reported a significant up-regulation of *IL-1*β and *IL-6* transcripts, especially after 3 h from LPS stimulus, compared to untreated cells (Fig. 4D and 4E); whereas a LPS-dependent increase of *TNF*-α mRNA levels occurred at early time (1 h) (Fig. 4F). Furthermore, differently from what observed in primary mouse HSCs we found that *α-SMA* and *COL1* mRNA levels remained unaltered under LPS addition (Fig. S4C), whereas *TGF-β* and *COL6* mRNA levels were significantly increased by 500 ng/ml LPS after 1 h of exposure (Fig. 4G).

**Figure 5:**
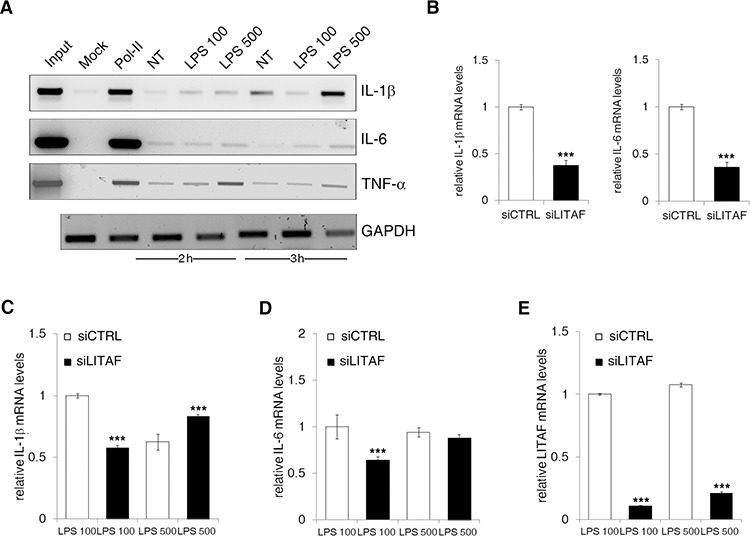
LITAF binds and regulates *IL-1*β and *IL-6* promoters **A.** LX-2 cells were exposed for 2 and 3 h to 100 ng/ml and 500 ng/ml LPS. Input, mock and mouse anti-RNA Pol II antibody were used as ChIP control. LITAF immunoprecipitated was used for the amplification by qPCR of *IL-1*β, *IL-6* and *TNF*-α promoters. Input DNA was used to assess *GAPDH* transcript for normalization (data presented are representative of three independent experiments). **B.** QRT-PCR analysis of *IL-1β* and *IL-6* in control (siCTRL) and LITAF-silenced (siLITAF) LX-2 cells. **C–E.** siCTRL and siLITAF LX-2 cells treated with 100 ng/ml and 500 ng/ml LPS. QRT-PCR of *IL-1*β (C), *IL-6* (D) and *LITAF* (E) mRNA in the presence of LPS at 3 h (*n* = 3). Histograms represent the mean ± SD. ***p < 0.001 for comparison between siCTRL and siLITAF cells, Student's *t* test.

Based on CTCCC repeats, the already described LITAF responsive element within *TNF*-α promoter [16], we designed specific primers for each cytokine promoter (Fig. S5A). Noteworthy, the results of chromatin immunoprecipitation (ChIP) indicated that LITAF specifically binds the promoter of all the three crucial pro-inflammatory genes, namely *IL-1*β, *IL-6* and *TNF*-α. In particular, LITAF displayed a more pronounced binding to these promoters just after 2 h from LPS stimulation (Fig. 5A). To investigate if *IL-6*, *TNF*-α and *IL-1*β are truly LITAF-targeted transcripts, LX-2 cells were transduced with either LITAF shRNA or nonspecific scRNA lentiviral plasmid. Next, cells were maintained in the presence of puromycin for more than 4 weeks for the establishment of stable silenced and control cell lines. The stable expression of LITAF shRNA caused a reduction of approximately 70% and 90% of LITAF protein and mRNA, respectively (Fig. S5B). Moreover, we found that the delivery of LITAF shRNA in LX-2 cells significantly reduced *IL-1*β and *IL-6* mRNA expression (Fig. 5B), but unaffected the levels of *TNF*-α, α-*SMA*, *TGF*-β, *COL1* and *COL6* transcripts (Fig. S5C), as well as platelet-derived growth factor receptor (PDGFR)-β mRNAs (Fig. S5D). Interestingly, LITAF silencing reduced the 100 ng/ml LPS-induced *IL-1*β and *IL-6* mRNAs after 3 h (Fig. 5C and 5D). Diversely, 500 ng/ml LPS was ineffective to produce similar reduction, probably due to a partial recovery of *LITAF* mRNA (Fig. 5E).

All this data confirmed that LITAF silencing can effectively interfere with the transcription of pro-inflammatory genes in HSC cell lines. We also reported a down-regulation of α-SMA protein expression in LITAF-silenced LX-2 cells compared to control (Fig. S5E), and the inability of LPS to recover this effect (Fig. S5F).

### p38MAPK is an upstream factor for LITAF nuclear translocation and activity

**Figure 6:**
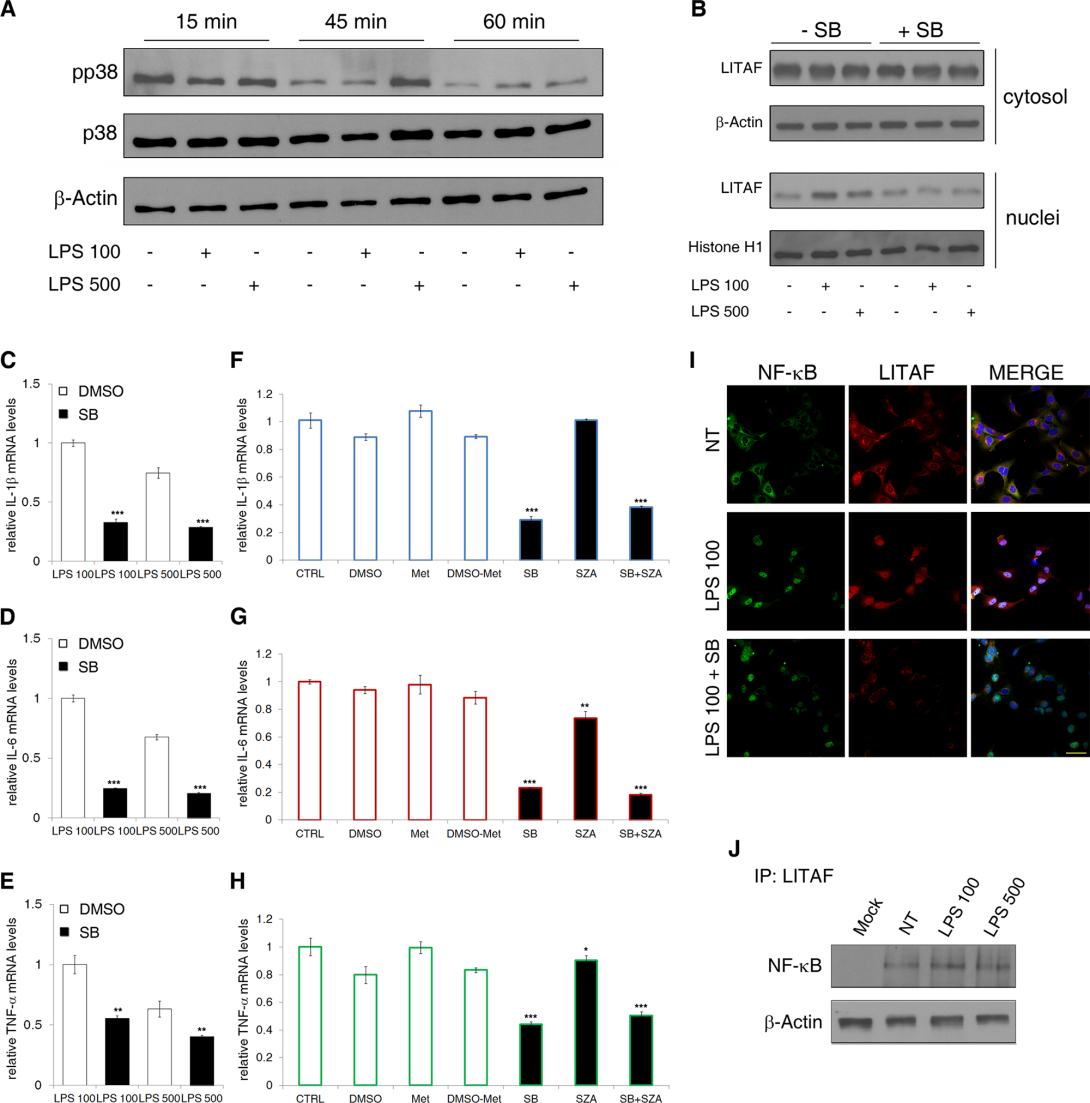
p38MAPK and p65NF-ĸB role in LITAF nuclear translocation and activity **A.** Immunoblot analyses showing the expression of Thr180/Tyr182 phosphorylated form (pp38) and total form (p38) of p38MAPK in LX-2 cells under 100 ng/ml and 500 ng/ml LPS treatment for 15, 45 and 60 minutes (data presented are representative of three independent experiments). **B.** Immunoblot analysis of cytosolic and nuclear LITAF after 30 minutes of SB203850 pre-treatment followed by 2 h of 100 ng/ml and 500 ng/ml LPS exposure (data presented is representative of three independent experiments). **C–E.** QRT-PCR analyses of *IL-1β* (C), *IL-6* (D) and *TNF-α* (E) mRNA after 30 min of SB203850 pre-treatment followed by 3 h of 100 ng/ml and 500 ng/ml LPS exposure (*n* = 3). Histograms represent the mean ± SD. **p < 0.01, ***p < 0.001 *vs.* vehicle (DMSO), Student's *t* test. **F–H.** QRT-PCR analyses of *IL-1*β (F), *IL-6* (G) and *TNF*-α (H) mRNA in LX-2 cells under LPS 100 ng/ml exposure pre-treated or not with single SB203850 and SZA or in combination (*n* = 3). Histograms represent the mean ± SD. *p < 0.05; **p < 0.01; ***p < 0.001 *vs.* vehicle (DMSO or Methanol), Student's *t* test. **I.** Representative confocal imaging of p65NF-ĸB (green), LITAF (red) and nuclei (blue) after 2 h from LPS treatment of LX-2 cells pre-treated or not with SB203850 (*n* = 3). **J.** Representative (*n* = 2) immunoprecipitation between LITAF and p65NF-ĸB. β-actin was probed as loading control.

Tang et al. [17] identified the TLR-4-dependent p38 mitogen-activated protein kinase (p38MAPK) as the responsible of LITAF phosphorylation/activation and nuclear translocation both in monocytic cells and in mouse macrophages. Hence, we investigated the p38MAPK expression and activation in LX-2 cells before and after the LPS treatment. We found that the expression of total p38MAPK was unaffected, whereas Thr180/Tyr182 phosphorylated form was significantly increased by LPS within 45 minutes of treatment (Fig. 6A). Furthermore, immunoprecipitation analysis showed that after 45 minutes from LPS addition the binding of LITAF to p38MAPK and its serine phosphorylation increased (Fig. S6A). As expected, SB203580, a specific inhibitor of p38MAPK, abolished LITAF nuclear translocation. After inhibitor dosage titration (data not shown), we found that only 30 μM SB203580 pre-treatment for 30 minutes reduced LPS-dependent LITAF nuclear translocation at 2 h in LX-2 cells (Fig. 6B and Fig. S6B).

To confirm the role of p38MAPK as upstream factor regulating LITAF-dependent transcriptional activity on pro-inflammatory genes we performed qRT-PCR analyses. We found that SB203580 reduced the mRNA expression of both *IL-1*β, *IL-6* and *TNF*-α under LPS addition (Fig. 6C–6E). This evidence was coupled with the SB203580 inhibitory effect on LITAF nuclear translocation and on LITAF binding to *IL-1*β, *IL-6* and *TNF*-α promoters (Fig. S6C), supporting the pivotal role of nuclear LITAF as pro-inflammatory transcription factor. We also found that both concentrations of LPS caused a significant increase of *TLR-4* mRNA (Fig. S6D). Moreover, the p38MAPK inhibition by SB203580 reduced LPS-dependent increase of *TLR-4* transcription (Fig. S6E). The latter result suggests that nuclear translocation of LITAF could be mediated by LPS/TLR-4-activated p38MAPK.

### LITAF functionally and physically interacts with NF-ĸB to regulate some pro-inflammatory genes

In order to define if the inhibitory effects of SB203850 on LPS-induced *IL-1*β, *IL-6*, *TNF*-α and *TLR-4* transcription could be exclusively associated to LITAF activity or if it could be the result of LITAF interaction with the master pro-inflammatory transcription factor NF-ĸB, we performed *in vitro* experiments on LX-2 cells with sulfasalazine (SZA). This drug blocks NF-ĸB-dependent transcription at micro- to milli-molar concentrations by the inhibition of IĸBα degradation [32]. We pre-treated LX-2 cells with 0.5 mM SZA, which inhibited LPS-induced NF-ĸB nuclear translocation (Fig. S6F). The same SZA concentration, alone or in combination with 30 μM SB203850, was used to treat LX-2 cells before 100 ng/ml LPS addition. We demonstrated that *IL-1*β transcription was down-sized only by p38MAPK inhibitor (Fig. 6F), whereas *IL-6* and *TNF*-α transcripts were mainly de-regulated by SB203850 with a minor effect of SZA (Fig. 6G and 6H). These results suggested that all the analysed cytokines could be mainly regulated by p38MAPK potential effects on LITAF activity and other downstream factors, such as the same p65NF-ĸB. Noteworthy, we found that, although p38MAPK inhibitor prevented LPS-induced nuclear translocation of LITAF, it was concurrently almost ineffective on that of p65NF-ĸB (Fig. 6I). Nevertheless, a functional and physical interaction between LITAF and p65NF-ĸB cannot be not excluded. In fact, siLITAF LX-2 cells under 100 ng/ml LPS exposure expressed a strongly reduced amount of p65NF-ĸB into the nucleus with respect to siCTRL counterpart (Fig. S6G). Moreover, we found that LITAF and p65NF-ĸB co-precipitated, and that this interaction was moderately susceptible to LPS addition (Fig. 6J). Further experiments are needed to elucidate the potential mechanisms involved in this cross-talk.

### Circulating levels of IL-1β correlate with LPS, the number of LITAF-positive HSCs and inflammation in paediatric patients with NAFLD

The activation of a chronic low-grade inflammation in NAFLD patients occurs in liver-resident cells, often reflecting tissue damage severity [33]. Therefore, we next set out to assess if circulating levels of LPS and cytokines correlated with LITAF expression in HSCs and histological features of NASH.

Plasma levels of IL-1β, IL-6, TNF-α and LPS were analysed in 40 children (male/female ratio 27:13) with biopsy-proven NAFLD and 8 controls (male/female ratio 5:3). The mean age of NAFLD patients was 12.0 years ± 2.6 (SD) and the mean body mass index (BMI) was 29.4 kg/m^2^ ± 10.6. Plasma levels of IL-1β, IL-6, TNF-α and LPS were significantly increased (*p* < 0.001) in NAFLD children as compared to the age-matched control subjects (Fig. S7A–S7D).

**Figure 7:**
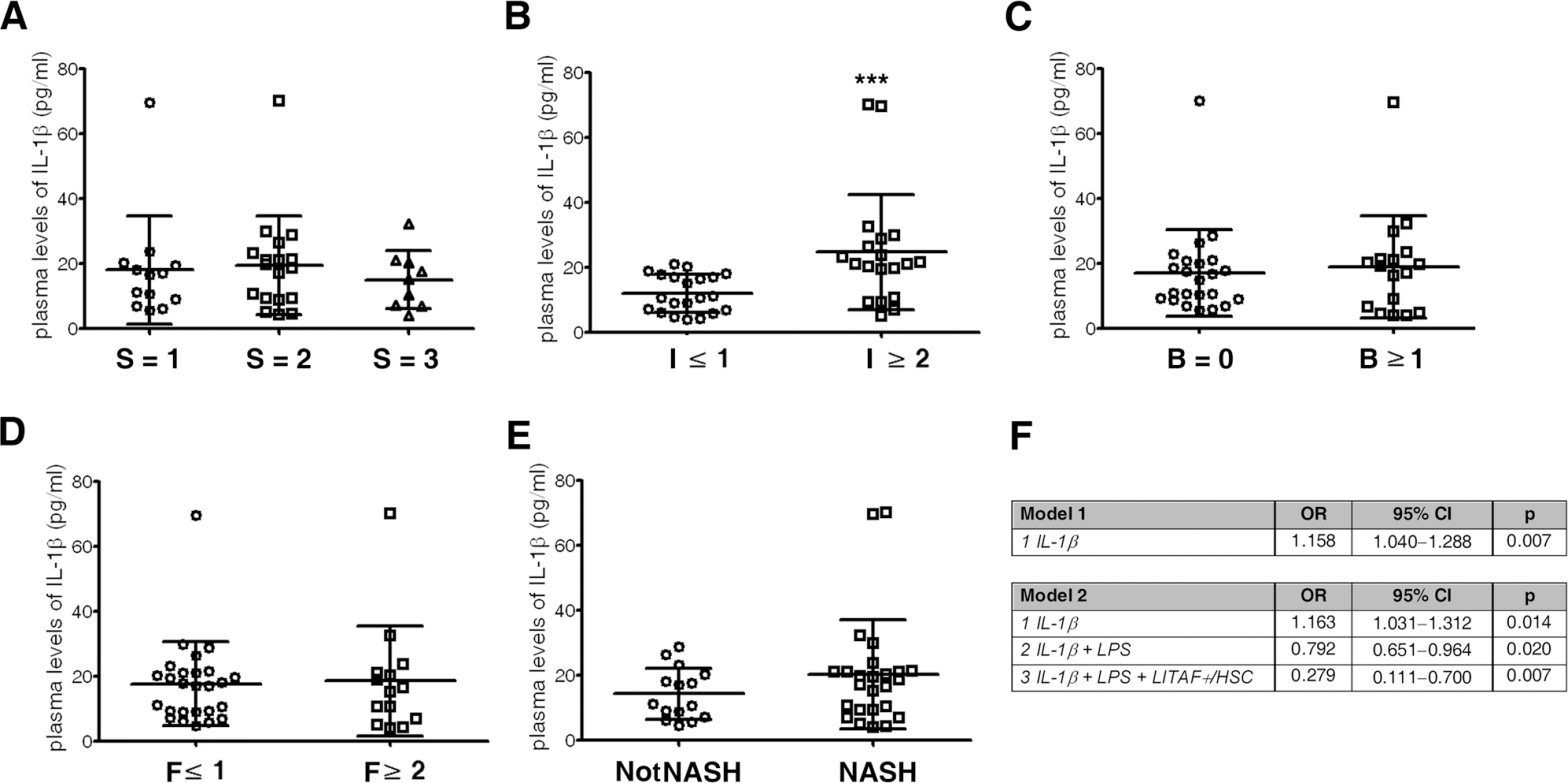
Circulating IL-1β acts as descriptor of inflammation and fibrosis in paediatric patients with NAFLD **A–E.** Scatter dot plots of plasma levels of IL-1β with respect to (A) steatosis grade (S = 1, S = 2, S = 3); (B) inflammation (I ≥ 2 *vs*. I ≤ 1); (C) ballooning (B ≥ 1 *vs*. B = 0); (D) fibrosis (F ≥ 2 *vs*. F ≤ 1); and (E) the presence of NASH (*n* = 40). Mean value ± SD. ***p < 0.001. Mann-Whitney *U* test. **F.** Odds ratios for inflammation (model 1) and fibrosis (model 2). The analyses was performed by including IL-1β IL-6, TNF-α, LPS and LITAF-positive HSCs.

Next, the correlations between circulating levels of IL-1β, IL-6, TNF-α and LPS, the number of LITAF-positive HSCs and the histological pattern of disease were assessed. The plasma levels of IL-1β were significantly correlated with those of LPS (Pearson's *r* = 0.62, *p* < 0.001), with the number of LITAF-positive HSCs (Pearson's *r* = 0.31, *p* < 0.05) and with inflammation (Pearson's *r* = 0.48, *p* < 0.01). Interestingly, the number of LITAF-positive HSCs significantly correlated (Fig. S7E) not only with IL-1β levels but also with inflammation (Pearson's *r* = 0.34, *p* < 0.05) and fibrosis (Pearson's *r* = 0.69, *p* < 0.001) and with the presence of NASH (Pearson's *r* = 0.66, *p* < 0.001). Further correlation data are also reported (Fig. S7E). Moreover, our data revealed that circulating levels of IL-1β were significantly (*p* < 0.001) increased in children with I ≥ 2 compared to subjects with I ≤ 1 (Fig. 7B). No statistically significant differences in IL-1β plasma levels were found to be in relation with steatosis grade (Fig. 7A), ballooning and fibrosis severity (Fig. 7C and 7D) and comparing NASH *vs*. NotNASH group (Fig. 7E). Interestingly, we also found that IL-1β levels in plasma correlated with those expressed in the liver (Pearson's *r* = 0.66, *p* < 0.01).

Finally, multinominal regression analysis revealed: in model 1 (Fig. 7F, upper panel) that plasma levels of IL-1β were significantly associated with inflammation, while the other variables, including IL-6, TNF-α, LPS and the number of LITAF-positive HSCs, were dropped as not significant; in model 2 (Fig. 7F, lower panel) that the IL-1β plasma levels were significantly associated with fibrosis, and forward stepwise inclusion of LPS and LITAF-positive HSC number variables positively influenced odds ratios.

## DISCUSSION

**Figure 8:**
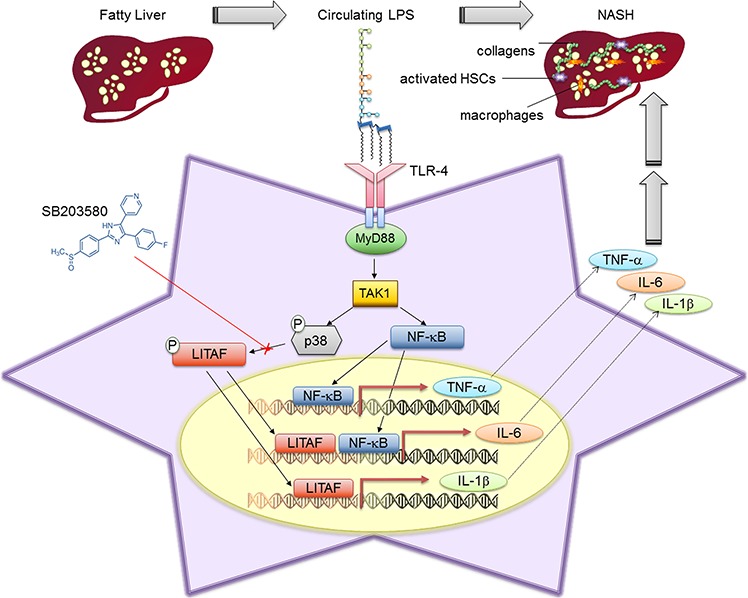
Schematic representation of LPS-induced *LITAF* transcription role in HSCs during NAFLD Activation of TLR-4 through binding of LPS leads to receptor dimerization and the recruitment of adaptor proteins, such as MyD88. This triggers the engagement of several other protein complexes resulting in the activation of TAK1. Once activated TAK1 may promote: 1) phosphorylation and activation of p38MAPK which supports the LITAF-dependent production of IL-1β and IL-6; 2) nuclear translocation and activation of p65NF-ĸB which regulates the production of TNF-α. SB203580 may hamper p38MAPK activation resulting in the inhibition of LITAF phosphorylation, nuclear translocation and transcriptional activity.

In this study, we show that LPS induces several pro-inflammatory cytokines *via* activation of LITAF in HSCs, resulting in a pattern of inflammation and fibrosis that may be responsible for a part of the tissue damage in NASH (Fig. 8). This is the first report to demonstrate that LITAF may be crucial in NAFLD pathogenesis by driving HSC responsivity to LPS.

The NAFLD has become the most common chronic liver diseases in both children and adults [34, 35]. NAFLD conditions range from simple steatosis to NASH, characterized by the presence of necrosis, inflammation and eventually fibrosis. The molecular mechanisms involved in the pathogenesis of NAFLD still remain unclear [36–41]. Previous studies suggest a plausible role for the gut-liver axis and of intestinal bacterial products, such as LPS, in NAFLD-related liver damage [42–46]. HSC activation is a key event in NAFLD, accounting for inflammatory molecule production and being the major player for matrix deposition during fibrogenesis [22, 26, 27, 30, 47, 48]. It is well known that HSCs express TLR-4, which, interacting with its principal ligand LPS, initiates a signalling cascade culminating in a pro-inflammatory and pro-fibrogenic response [49, 50]. Several lines of evidence have demonstrated that low-to-high doses of LPS induce synthesis and release of pro-inflammatory cytokines in human and rodent models of quiescent and transitionally activated HSCs [7, 51–53].

We previously demonstrated a strong correlation between increased circulating levels of both LPS and TNF-α and liver damage in paediatric patients with NAFLD [5]. We also reported a significant alteration in protein/mRNA expression levels of LITAF in rats with diet-induced NAFLD [19]. Here we show that an increased expression of LITAF protein in parenchymal and non-parenchymal liver correlates with the severity of the disease in children with NAFLD, confirming our previous data in the rat model. Furthermore, we observed that hepatocellular LITAF over-expression occurs mainly at the interface with the fibrotic area where α-SMA reaches its maximal expression. The reasons of this phenomenon recall further exploration. This finding suggests that LITAF may play an important role in determining liver damage in NAFLD. In particular, the upsized intra-hepatic expression of LITAF was observed in subjects that displayed a more severe pattern of inflammation and fibrosis. Although all liver-resident cells are involved in the development of NAFLD and its progression to NASH, activated HSCs are the main cell type responsible for fibrogenesis in the liver. Activated HSCs are known to produce several pro-inflammatory mediators that contribute to maintain themselves activated and capable of exacerbating hepatic fibrosis and inflammatory milieu [23]. Therefore, here we tried to dissect the role of LITAF in the pro-fibrogenic and pro-inflammatory phenotype of HSCs. Our data indicates that LITAF can be an LPS-induced pro-inflammatory and pro-fibrogenic mediator during NASH development and suggests that this transcription factor contributes to the liver damage caused by HSC activation. Interestingly, LITAF is mostly nuclear in activated HSCs suggesting a switch on of its transcriptional activity in these cells as demonstrated by Tang et al in macrophages [17]. It is well established that the status of HSC activation and its maintenance rely on the presence of different cytokines [54]. Our data shows that the maintenance of a serum-dependent activation state in HSCs is characterized by an enhanced repertoire of pro-inflammatory cytokines and chemokines of which IL-1β, IL-6 and TNF-α have been the focus of major interest. Mouse HSCs were found to produce these and other cytokines and chemokines under LPS stimulation. Using an extensive assortment of cytokines allowed us to confirm and amplify the pro-inflammatory response to LPS [7, 52]. Analysis of the LPS-induced pro-inflammatory and pro-fibrogenic pattern in LX-2 cells, which is the best characterized model of *in vitro* HSCs, has highlighted a similarity with primary mouse HSCs in concert with an up-regulation of LITAF transcription and expression into the nucleus. Our results are in agreement with previous data [52] showing the ability of LPS to promote pro-inflammatory more than pro-fibrogenic genes, and its anti-proliferative effect. We found within the *IL-1*β and *IL-6* promoters, a LITAF consensus sequence (CTCCC) already identified in *TNF*-α [16]. ChIP analysis confirms the LPS-dependent increase of LITAF binding to *IL-1β*, *IL-6* and *TNF*-α promoter region. Furthermore, we provide the first evidence that LITAF silencing in LX-2 HSCs reduces expression of both *IL-1*β and *IL-6* mRNAs but has no effect on *TNF*-α mRNA. Moreover, LITAF silencing is detrimental for low-dose LPS-mediated induction of *IL-1*β and *IL-6* transcripts, indicating that they are LITAF-dependent genes in HSCs activated towards a pro-inflammatory phenotype. Interestingly, in the same cells, LITAF silencing also affects the α-SMA protein expression suggesting no transcriptional activity effect but plausibly other post-translational regulatory mechanisms. Because endosomal fraction of LITAF interacts with ubiquitin ligases sequestering them into the lysosomes (e.g. Itch) [53], and given that α-SMA is described as one of the ubiquitinated proteins (mUbiSiDa: http://222.193.31.35:8000/detail_info.php?name=Q13707), LITAF depletion may enhance targeted proteins degradation. Accordingly, we found an up-regulation of ubiquitin expression in siLITAF cells (data not shown), but this mechanism requires further experimental exploration.

Thirunavukkarasu et al [54] have demonstrated that endotoxin-induced expression of TNF-α and IL-6 in HSCs is initiated by the p38MAPK and is mediated by NF-ĸB. However, p38MAPK, recognized as an upstream factor of LPS/TLR4/MyD88 signalling cascade, has been reported to regulate nuclear translocation and activity of pro-inflammatory transcription factors, including AP-1 and LITAF [17, 55]. Our data confirms an early activation of p38MAPK and its physical interaction with LITAF in LPS-treated LX-2 cells. The major role of LPS-activated p38MAPK on LITAF activity is validated by the inhibitory effects of SB203850 on LITAF nuclear translocation and transcriptional activity. The use of SB203850, alone or in combination with the NF-ĸB inhibitor SZA, allows us to exclude the possibility that p65NF-ĸB has a role in the regulation of *IL-1*β promoter, but does not rule out its potential cooperation with LITAF in regulating *IL-6* and *TNF*-α gene transcription. In fact, we demonstrated a physical interaction of LITAF with p65NF-ĸB in LX-2 cells.

The discovery that LITAF is a main regulator of IL-1β in HSCs could provide a new mechanism that may improve both our understanding of how NAFLD-related liver damage (i.e. inflammation and fibrosis) develops, and the design of novel therapeutic approaches. In fact, Kamari et al recently demonstrated [56] that IL-1β selective deficiency in liver parenchymal cells protected mice from diet-induced NASH and fibrosis. The relevance of this novel LPS/TLR-4/LITAF signalling in regulating potential development of hepatic inflammation and fibrosis that characterizes NASH is emphasised by the fact that children affected by this disease frequently display also a pattern of low-grade systemic inflammation. Furthermore, plasma levels of IL-1β avatar the mRNA hepatic expression of the same cytokine, suggesting a relevant contribution of liver cells to systemic increase of this inflammatory molecule. From a mechanistic viewpoint, these results suggest that LPS/LITAF pathway, regulating IL-1β production, may interact with the NLRP3 inflammasome complex that, sensing LPS, may induce IL-1β maturation [57, 58]. From a translational point of view, circulating and hepatic IL-1β, which associate to LPS and LITAF-positive HSCs, may outline a panel of potential biomarkers of more severe inflammation and fibrosis in children with NAFLD. Furthermore, in this setting it is conceivable that a targeted approach for preventing LITAF activity could mitigate NAFLD-related liver damage by reducing both hepatic and systemic IL-1β production/release. Unfortunately, currently no direct antagonist of LITAF activity exist, while several pharmaceutical companies have developed p38MAPK inhibitors for therapeutic use. Some of these drugs are in phase I and phase II clinical trials [59] and our findings suggest that they may virtually improve liver damage in patients with NASH.

In conclusion, the discovery of LITAF as a key regulator of pro-inflammatory and pro-fibrogenic response in HSCs provides new insights in the molecular pathogenesis of NAFLD-related damage. It also raises a series of provocative questions about the cell-to-cell communication “in and outside” the liver in patients with NAFLD, suggesting that IL-1β could be a common trigger for both progression of liver disease and development of co-morbidities.

## MATERIALS AND METHODS

### Human subjects and liver histology

Cohort 1 included 8 children with no evidence of fatty liver at ultrasonography and subjected to appendicectomy. Liver tissues from these patients were used for immunohistochemical/immunofluorescent analyses. Cohort 2 included 25 children with biopsy-proven NAFLD. Cryopreserved liver tissues from these patients were used for molecular analyses. Cohort 3 included Cohort 2 samples and further 15 liver samples from children with biopsy-proven NAFLD. Blood samples and liver specimens (paraffin-embedded tissues) from these patients were used for the analysis of circulating markers and immunofluorescence. Cohort 4 included Cohort 1 samples and further 17 liver samples from children without liver disease. All samples were collected at the Hepato-Metabolic Disease Unit of the Bambino Gesù Children's Hospital between March 2013 and September 2014. Main inclusion criteria were complete abstinence from alcohol and the absence of other liver diseases. Diagnosis of NAFLD was performed on bioptic samples, fixed in 10% buffered formalin and processed for hematoxylin and eosin staining and Masson's trichrome staining. Grade and stage of disease were assessed by the NAFLD Clinical Research Network criteria [21].

### Animals and HSC isolation

Adult, male Balb/c mice were provided by Dr. Svegliati Baroni's Lab (Ancona, Italy). The animals were permitted *ad libitum* consumption of water and standard rodent food. Upon completion of 8 weeks of treatment, mice were killed and primary mouse HSCs isolated from normal liver. The isolation procedure was performed by collagenase-pronase perfusion technique followed by density gradient centrifugation on Nycodenz gradients as described previously [41]. After purity and viability check, the isolated cells were seeded on plastic dishes and chamber slides at a density of 2 × 10^5^/ml and cultured in DMEM supplemented with 10% foetal bovine serum (FBS) and penicillin/streptomycin as described previously. Cells were cultured for 5 days in medium supplemented with 10% FBS and then serum starved for additionally 24 hours. Cells were then stimulated with LPS (100 and 500 ng/ml) in the presence of either 1% (HSC-1) or 10% (HSC-10) FBS. Cells were then collected at different timepoints (1, 2, 3, and 6 h).

### Human LX-2 cell line

The human LX-2 cell line, purchased by Merck Millipore (Germany), were seeded into 6-well plates with 10^5^ cells per well in DMEM supplemented with 10% FBS, 2 mM L-glutamine, 100 units/ml penicillin, and streptomycin at 37°C in 5% CO2. At about 60–70% confluence two different concentrations of LPS (100 or 500 ng/ml) were added to the medium and cells were harvested at different timepoints (15 and 45 min, 1, 2, 3, 6 and 24 h) from the treatment. PBS was added as vehicle control. When requested, a 30 min pre-treatment was performed before the LPS stimulus with 30 μM SB203580 (Sigma-Aldrich Inc. MO, USA) and/or 0.5 mM sulfasalazine (Sigma-Aldrich Inc. MO, USA). DMSO and/or Methanol were added as vehicle controls.

### Lentiviral shRNA silencing of LITAF in LX-2 cells

Lentivirus-delivered stable silencing was carried out on LX-2 cells seeded in 6-well plates at 80% confluence. Cells were incubated with the lentiviral particles (multiplicity of infection, MOI = 4) in presence of hexadimethrine bromide (8 μg/ml). Lentiviral particles used for LITAF silencing were Mission shRNA TRCN0000297833 and TRCN0000280440 (SHCLNV, Sigma-Aldrich Inc. MO, USA). As a control, LX-2 cells were also transduced with viral particles (MOI = 4) containing non-targeting shRNA, namely MISSION^®^ pLKO.1-puro Non-Target shRNA Control Transduction Particles (SHC002V, Sigma Aldrich Inc. MO, USA). Transduced cells were selected in complete growth medium containing puromycin (5 g/ml). The silencing efficiency was monitored both by qRT-PCR and immunoblot analysis.

### Immunofluorescence

Immunofluorescence was performed on 2 μm sections of paraffin-embedded liver tissue and on LX-2 and primary mouse HSCs seeded on 4-well chamber slides and fixed in cold methanol/acetone (2:1). The slices were incubated with primary antibodies (listed in Table S1). Detection of the primary antibodies was performed using 1:500 Alexa Fluor^®^ conjugated secondary antibodies (Invitrogen/Molecular Probes Corp, CA, USA). The images were captured and analyzed using Olympus FluoView FV1000 confocal microscope. The images were acquired using an identical acquisition time for all tissue sections.

### Double staining: immunohistochemistry and immunofluorescence

Immunostaining of formalin-fixed paraffin-embedded tissue specimens was performed on 2 μm sections. After dewaxing and rehydrating, heat-induced epitope retrieval was performed by boiling the slides with Dako Target Retrieval Solution pH 8 (10X). Endogenous peroxidase was blocked with 3% hydrogen peroxide followed by incubation with avidin/biotin blocking system (Thermo Scientific, IL, USA) to inhibit endogenous biotin activity. We used primary polyclonal rabbit antibodies raised against LITAF. Staining was performed with Dako Envision Plus System (Dako Cytomation, Italy). Once developed the staining for LITAF by 3, 3′-diaminobenzidine (DAB) Substrate (Vector Laboratories, CA, USA), the double staining was performed by a second labeling via immunofluorescence technique. The slides were incubated again for 1 h with 5% BSA to block eventual non-specific sites. Subsequently, the sections were incubated with the second primary antibody, the mouse monoclonal α-SMA diluted in 5% BSA in PBS, at +4°C overnight. A secondary antibody anti-mouse Alexa Fluor^®^ 488 dye (1:500, Life Technologies, CA, USA) was used. The nuclear staining was carried out by 5 minutes incubation with 2′-(4-hydroxyphenyl)-5-(4-methyl-1-piperazinyl)-2, 5′-bi-1H-benzimidazole dihydrochloride hydrate (Hoechst) (Sigma-Aldrich Inc. MO, USA) diluted 1:10000 in 1X PBS. The slides were finally mounted with aqueous base 60% glycerol. For primary antibodies details, see Table S1. Confocal imaging was performed using an Olympus Fluoview FV1000 confocal microscope equipped with FV10-ASW 2.0 software, Multi Ar (458–488 and 515 nm), 2X He/Ne (543 and 633 nm), 405-nm diode lasers and a 60x 1.42 oil objective. Optical sections were acquired with a format of 1024 × 1024 pixels, a sampling speed of 40 μs/pixel, and 12 bits/pixel images. Fluorochrome unmixing was performed by automated-sequential collection of multi-channel images to reduce spectral cross-talk between channels.

### Automatic quantification and comparison of nuclear LITAF concentration

In total, images from 253 slides were acquired with the Olympus Fluoview FV1000 confocal microscope at 400x resolution, and stored as 32 bit RGB images of 1024 × 1024 pixels. Each image includes the two levels (channels) corresponding to nucleus and LITAF staining.

A computational pipeline was developed to quantify the amount of LITAF within cell nuclear regions in the digitalized images (Fig. S2A). The pipeline first localizes nuclear regions in the nuclear-stained channel (steps 1–6), and then estimates the nuclear LITAF concentration by considering the intensity in the LITAF staining channel of the pixels within the nuclear regions localized in the nuclear-stained channel (step 7).

More in details, cell nuclei are localized by applying the following procedure. Images are first preprocessed with the Otsu thresholding algorithm [60] to remove the background noise (step 1). To take into account the differences in illumination and staining across the slides, which would bias the standard Otsu thresholding leading to false negatives in the detection of nuclei, we applied a local Otsu method. Briefly, each image was divided in a grid of nine blocks, and the Otsu method was applied independently within each block, producing a (potentially) different threshold for each block. Then, a pixel-by-pixel local threshold was calculated as the average of the nine thresholds, weighted by the distance from the centroid of each block to the pixel.

After thresholding, the gaps within nuclear regions were filled by convolving each thresholded image with a Gaussian diffusion process (step 2), and applying the erosion morphological operator to the result of the convolution (step 3). This procedure also removes the skewness from nuclear borders. The Watershed algorithm [61] was then used to segment the resulting image and localize each nucleus (steps 4–5). Finally, a post-processing step was applied to removing segmented regions that were too small to represent nuclei (step 6).

For each nucleus, the median of the corresponding pixel intensity in the LITAF staining channel was used as estimate of the nuclear LITAF concentration (step 7). For each biological sample, the median of the nuclear LITAF concentration of all the nuclei was considered as nuclear LITAF concentration index. The computational pipeline has been implemented in Python 2.7, relying on morphological operators and watershed algorithms from the Mahotas library [62].

### RNA isolation and Quantitative Real-time PCR

Total RNA was isolated from cells using TRIzol (Invitrogen, Carlsbad, CA, USA) and from liver tissues by RNA Purification Plus Kit (Norgen Biotek Corporation, ON, Canada) following manufacturers' instructions. cDNA was synthesized by High Capacity cDNA Reverse Transcription Kits (Applied Biosystems, Foster City CA, USA) according to kit's instructions. Quantitative Real-Time (qRT-PCR) amplification, detection and analysis was performed by ABI Prism 7900HT Fast Real-Time PCR System (Applied Biosystems, Foster City CA, USA) using TaqMan^®^ Fast Universal PCR Master Mix (2X) No AmpErase^®^ UNG (Applied Biosystems, Foster City CA, USA). *GAPDH* housekeeping gene was used as a reference control for normalization. Based on the ΔΔCt method, relative amounts of mRNA were expressed as fold changes versus controls. Primer references are listed in Table S2.

### Immunoprecipitation and western blotting

Cells were collected and lysed with Ripa Buffer. Whole cell extracts were quantified using the BCA™ Protein Assay (Thermo Scientific, IL, USA). For immunoprecipitation, approximately 0.5 mg of the lysate protein was immunoprecipitated using 1 μg of rabbit polyclonal anti-LITAF antibody (sc-66945 Santa Cruz Biotech. CA, USA) at 4°C overnight and next incubated with protein A/G-agarose (Santa Cruz Biotech. CA, USA) at 4°C for 1 h. Whole lysates and eluates were prepared in sample buffer and separated by SDS-PAGE resolving gels. Proteins were electrophoretically transferred to Hybond-C Extra membranes (Amersham, Germany) and incubated firstly with primary antibodies, then with HRP-conjugated secondary antibody (Santa Cruz Biotech. CA, USA) following standard methods [63]. Protein expression was quantified by densitometry analysis using Image J v3.91 software. For primary antibodies details, see Table S1.

### Cytokine protein array analysis

Mouse Inflammation Antibody Array C1 (Cat# AAM-INF-1) purchased from RayBiotech, Inc. (Norcross, GA, USA) was employed to evaluate the expression of 40 different cytokines in cell lysates from primary mouse HSC-10 and HSC-1 in presence or absence of LPS stimulus. The assay was carried out according to the manufacturer's directions. Array membranes, spotted in duplicate with high quality capture antibodies, were incubated with 2 ml of Blocking Buffer at room temperature for 30 min. The membranes were incubated with 200 μg of protein lysate from each sample (diluted 5 fold with 1X blocking buffer) at room temperature for 2 h. After three time washing with Wash Buffer I and three time washing with Wash Buffer II, a working solution of Biotinylated Antibody Cocktail was added to the membrane at 4°C overnight. Then, the membranes were washed as previously described and further incubated at room temperature for 2 h with diluted HRP-conjugated streptavidin solution. After washing as previously described, a mix of Detection Buffer C and D (1:1) was used as recommended by instructions provided by the manufacture protocol. Finally, the chemiluminescent signal was acquired by ChemiDoc™ MP Imaging System (Bio-Rad, Italy) and densitometry of the specific spots was performed with ImageJ v3.91 software. The results were expressed as relative protein expression of individual normalized signals of A-HSCs sample compared with that of NA-HSCs, treated or not with LPS.

### Cell proliferation assay

LX-2 cells were seeded at a density of 6–8 × 10^3^ in 96-well plates and then cultured for 3 and 24 h with or without LPS (100 and 500 ng/ml). The DELFIA (Dissociation-Enhanced Lanthanide Fluorescent Immunoassay) Cell Proliferation Assay was performed following the manufacturer instructions (PerkinElmer, MA, USA). Specifically, after LPS stimulus LX-2 cells were incubated with BrdU for 3 h. The europium-labeled antibody was used to detect incorporated BrdU following Delfia Cell Proliferation Assay protocol. The dissociation of europium ions from the anti-BrdU antibody and the formation of their fluorescent chelates were obtained by DELFIA inducer reagent. The fluorescence, which is proportional to DNA synthesis, was measured by time-resolved fluorometer 2100 Envision™ Multilabel Reader (PerkinElmer, MA, USA).

### Biochemistry

Plasma LPS was measured using a commercial available Limulus amoebocyte lysate chromogenic endpoint assay (Hycult Biotechnology, The Netherlands) suitable for detection of a concentration range from 0.01 to 10 EU/mL. The plasma levels of IL-1β, IL-6 and TNF-α were measured using human ELISA kit (BioVendor, Heidelberg, Germany).

### Chromatin immunoprecipitation assay

LX-2 cells were grown to 80%-90% confluence in 100 mm culture plates. After treatment the culture medium was removed and cells were cross-linked with 1% formaldehyde for 15 min at room temperature on rotating shaker platform. The Protein-DNA cross-linking reaction was stopped by adding glycine to a final concentration of 125 Mm. After 5 min of glycine incubation, the cells were washed twice with 1X PBS and collected in 1X PBS by cell scraper and subsequent centrifugation (3000 xg for 5 min). For each sample the cell pellet obtained was lysed with 1 ml of Lysis Buffer containing 1X protease inhibitors cocktail and sonicated by using a Branson Sonifier 450 (Branson, Danbury, CT, USA). To obtain DNA fragments of 300–1000 base pairs (bp) in length the following settings were used: 80% duty, 10 cycles of 15 pulses with 2 min cooling (ice/water). The DNA fragment sizes were checked by loading 5 μl of each sonicated product on a 1% agarose gel. For each immunoprecipitation 500 μg of soluble chromatin was diluted to a final volume of 1 ml with Lysis Buffer and centrifuged at 20000 × g to remove debris. To reduce unspecific binding, chromatin solution was pre-cleared by adding 50 μl pre-blocked Protein A agarose beads. Pre-cleared chromatin was immunoprecipitated by overnight incubation at +4°C with 5 μg of specific rabbit polyclonal anti-LITAF (sc-66945 Santa Cruz Biotech. CA, USA) and mouse monoclonal RNA polymerase II antibody (05–952 Merck Millipore, Germany) as positive control, while no antibody was added for the background control (MOCK).

The resulting immune-complexes were captured by 50 μl of pre-blocked Protein A agarose beads addition. The supernatants of each sample were saved as input (for positive control and data normalization). The agarose immunocomplexes were washed consecutively for 5 min on a rotisserie shaker with 1 ml of each solution: Lysis Buffer (50 mM Tris-HCl pH 8.0, 5 mM EDTA, 200 mM NaCl, 0.5% NP-40), High Salt Buffer (20 Mm Tris-HCl pH 8.0, 500 mM NaCl, 0.5% NP40, 0.05% SDS, 2 mM EDTA), Lithium Salt Buffer (10 mM Tris, pH 8.0, 250 mM LiCl, 1% NP40, 1% Deoxycholic acid) and TE buffer (10 mM Tris HCl pH 8.0, 1 mM EDTA), between each wash the supernatant was removed by centrifugation for 5 min at 3000 × g. After the last wash, the immune-complexes were eluted by adding 120 μl of Elution Buffer (20-min incubation at room temperature on a rotisserie shaker). DNA crosslink was reverted by heating at 65°C. After proteinase K digestion the immunoprecipitated DNA was extracted by phenol-chloroform method and the promoter regions of interest were amplified by PCR. The PCR primers (Sigma-Aldrich Inc. MO, USA) were designed to amplify DNA sequences encompassing LITAF consensus sites within *IL1*-β, *IL-6* and *TNF*-α promoters (for primer sequences used see Fig. S5A). PCR primers specific for GAPDH were used for normalization (forward 5′-GATGACATCAAGAAGGTGGTG-3′ and reverse 5′-CATACCAGGAAATGAGCTTG-3′).

### Statistics

Data were expressed as mean ± SD. Normality was evaluated by Shapiro-Wilk test. We planned to analyze differences across groups by Student's two-tailed (2-group comparison) or ANOVA (≥3 group comparison), when variables were normally distributed, or with Mann-Whitney *U* test (2-group comparison) or Kruskal-Wallis test (≥3 group comparison) where variables were non-normally distributed. Differences were considered statistically significant at *p* < 0.05.

Univariate analysis was performed by Pearson's correlation coefficient, after log-transformation of skewed data.

For liver histology, predictors of fibrosis (presence *vs*. absence) and of inflammation (presence *vs*. absence) were assessed by multivariate logistic regression analysis. For logistic regression analysis, continuous variables were divided in quartiles (STATISTICA software, 5.1, Statsoft Italia, Padua, Italy).

### Study approval

The study was approved by Ethical Committee of the Bambino Gesù Children's Hospital and written consent was obtained from the parents of the children (Protocol number 768.12). Animal experiments and cell isolation studies were performed according to the guidelines of the Ancona University Institutional Animal Care and Use Committees.

## SUPPLEMENTARY FIGURES AND TABLES


