# Supplementary material for: LPS-induced TNF-α factor mediates pro-inflammatory and pro-fibrogenic pattern in non-alcoholic fatty liver disease
Source: Oncotarget. 2015 Oct 8;6(39):41434–52. doi: 10.18632/oncotarget.5163 (PMC4747165; doi:10.18632/oncotarget.5163)
Supplement: Supplementary file 1 [file oncotarget-06-41434-s001.pdf]

SUPPLEMENTARY FIGURES AND TABLES

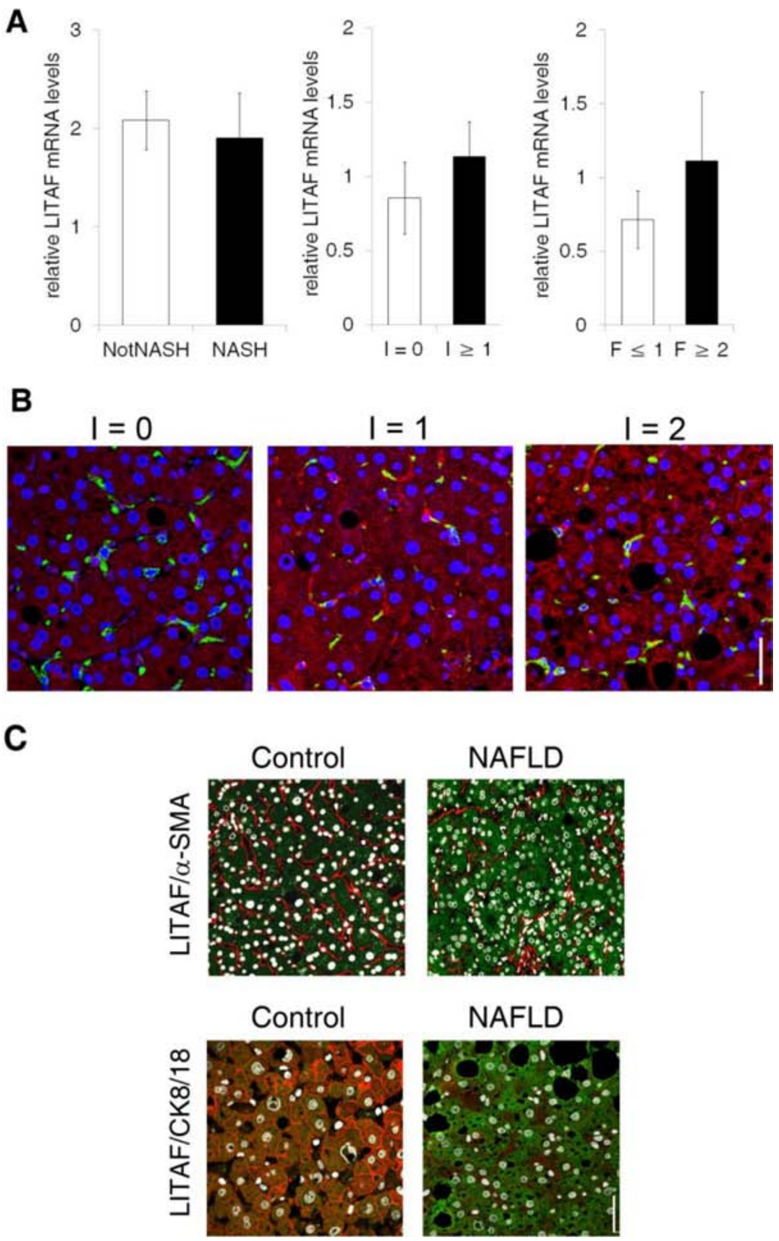

Supplementary Figure S1: LITAF protein expression increases in parenchymal and non-parenchymal liver cells of children with NAFLD. A. QRT-PCR analysis of *LITAF* in patients with NASH *vs*. NotNASH, with I ≥ 1 *vs*. I = 0 and with F ≥ 2 *vs*. F ≤ 1. Histogram represents the mean values ± standard deviation (SD) ($n = 25$). **B.** Representative confocal image of CD163 (red), LITAF (green) and nuclei (blue) staining (scale bar: 100 μm) performed in 25 liver tissues from children with NAFLD. **C.** Representative confocal laser microscopy of CK8/18 or α-SMA (red), LITAF (green) and nuclei (white) staining (scale bar: 100 μm) in liver tissues from 25 NAFLD children and 8 healthy controls.

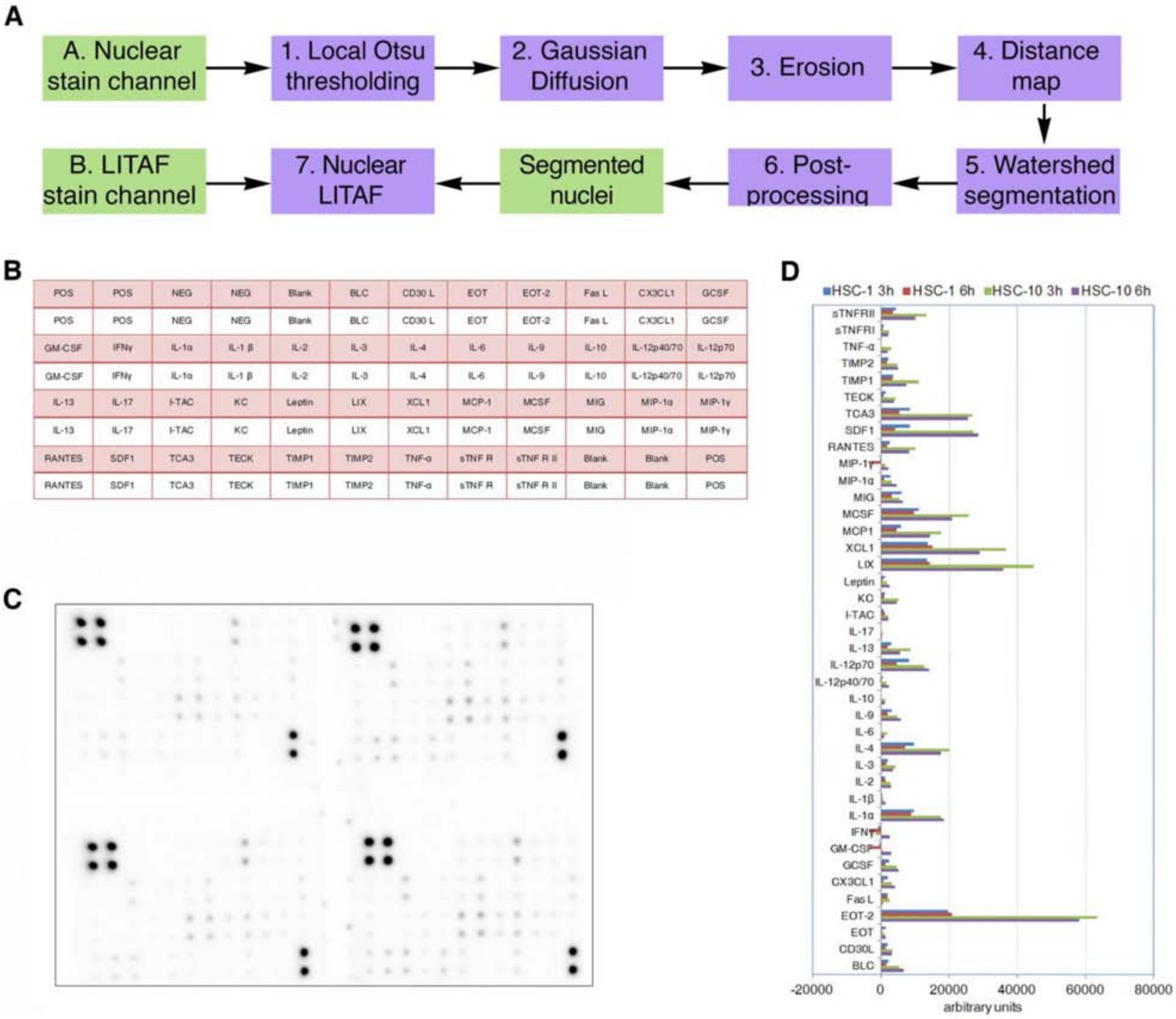

Supplementary Figure S2: Activated primary mouse HSCs display an increased serum-mediated expression profile of pro-inflammatory cytokines. A. Bioinformatic pipeline for quantitative analysis of imaging data. **B.** Layout of the antibodies as spotted on the membrane. The abbreviated names for 40 different cytokine probes are reported. **C–D.** Primary mouse activated HSCs treated with 1% FBS (HSC-1) or with 10% FBS (HSC-10) at different timepoints. C: Representative photographs of cytokine arrays in which each cytokine is represented by duplicate spots on a single membrane. D: The complete cytokine expression profile is reported as the mean of fold inductions of HSC-10 *vs*. HSC-1 at 3 h and 6 h ($n = 2$). Statistics is not reported.

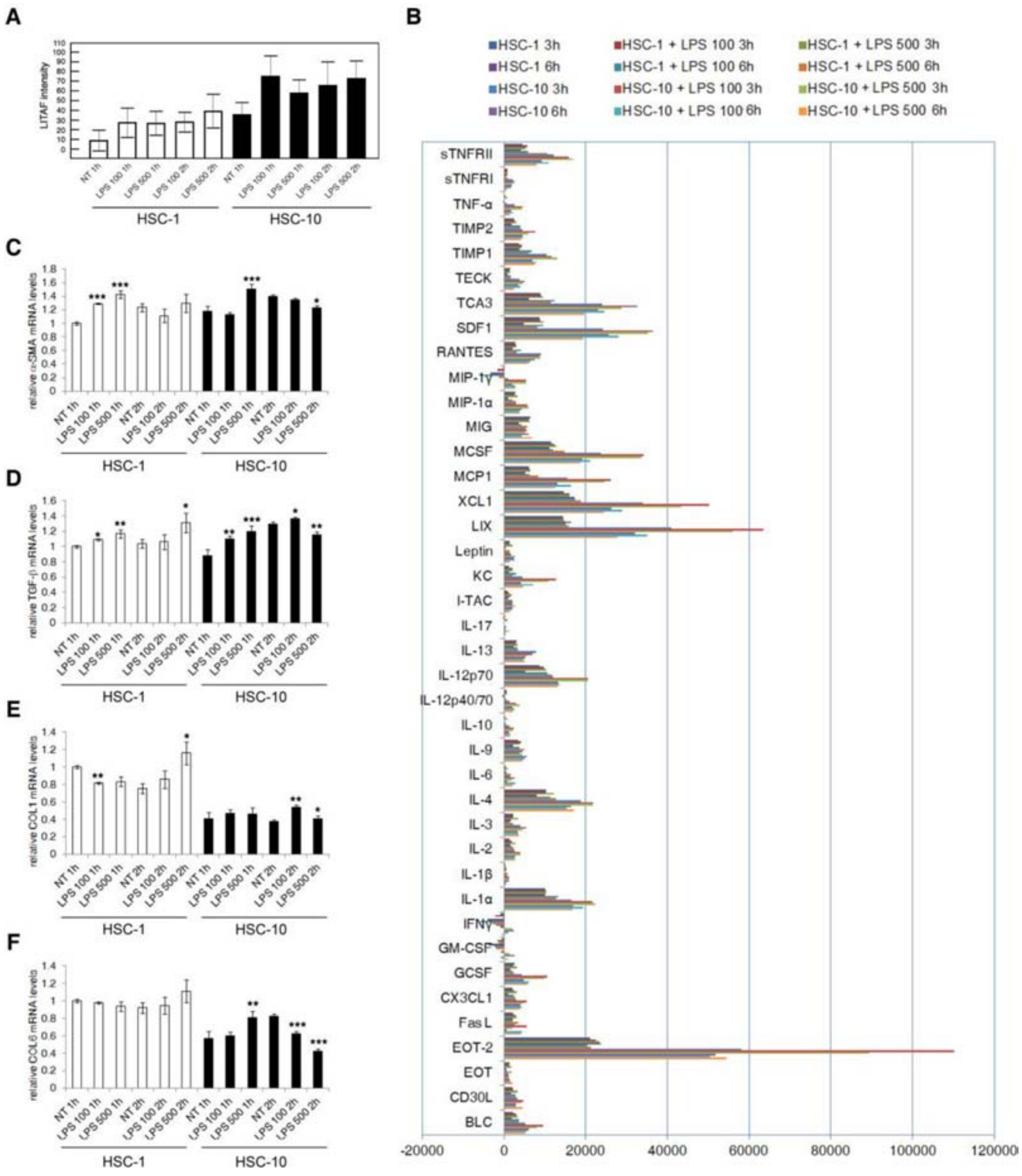

Supplementary Figure S3: LPS treatment increases pro-inflammatory cytokine expression profile in activated primary mouse HSCs. A–F. Primary mouse activated HSCs treated with 1% FBS (HSC-1) or with 10% FBS (HSC-10) after LPS treatment. A: Quantitative imaging analysis of LITAF nuclear translocation after 1 h and 2 h from LPS addition. Mann-Whitney *U* test. B: The complete cytokine expression profile is reported as the fold induction of HSC-10 *vs*. C–F: HSC-1 after 3 h and 6 h of treatment with or without LPS. QRT-PCR analyses of (C) *α-SMA*, (D) *TGF-β*, (E) *COL1* and (F) *COL6* after 1 h and 2 h from LPS addition ($n = 3$). Histograms represent the mean ± SD. $^*p < 0.05$ $^{**}p < 0.01$; $^{***}p < 0.001$ *vs*. NT, Student's *t* test.

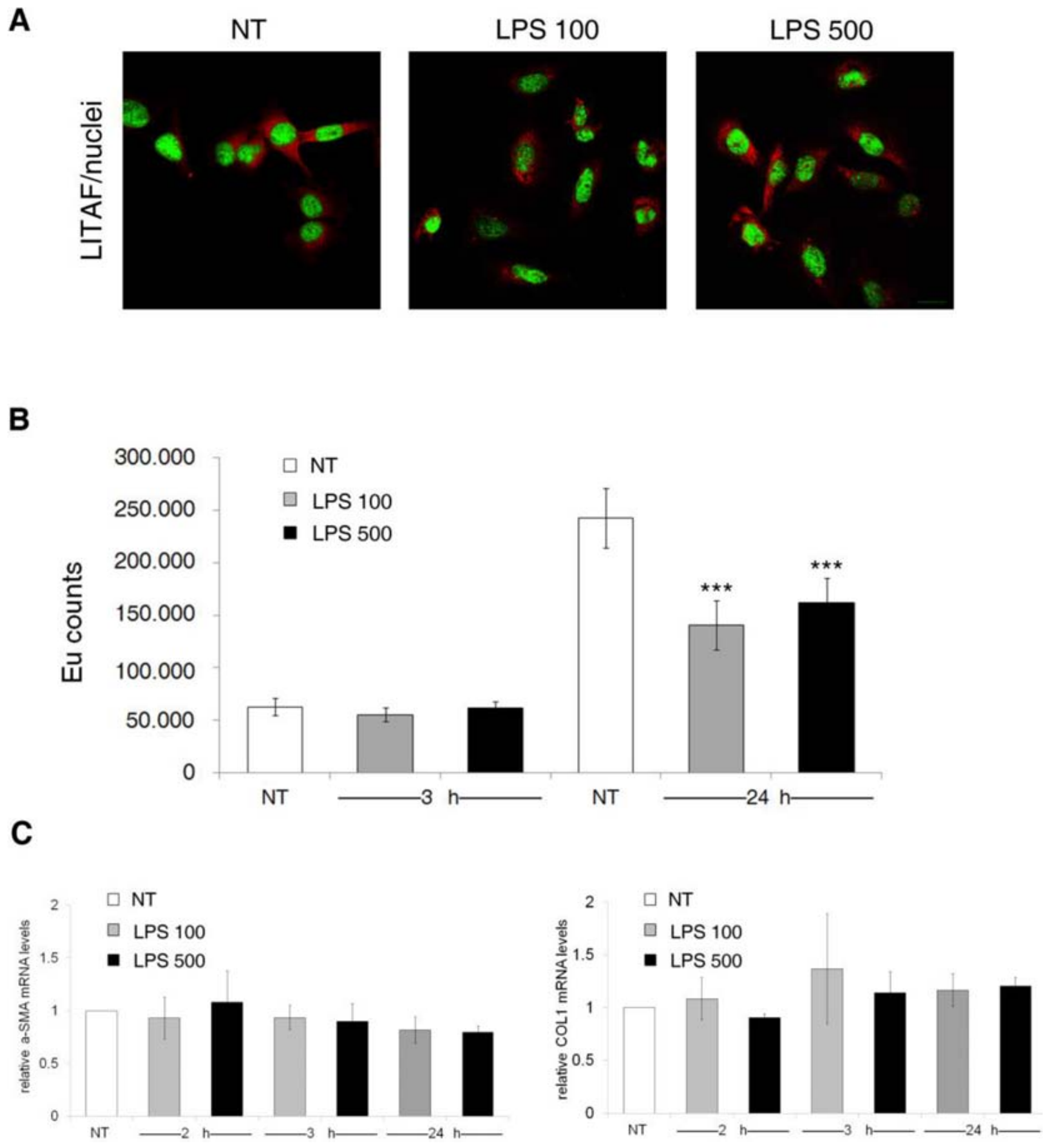

Supplementary Figure S4: LPS reduces LX-2 proliferation rate. A–C. LX-2 cells with (LPS 100 and LPS 500) or without (NT) LPS treatment. A: Confocal laser microscopy analysis of LITAF (red) and nuclei (pseudocoloured in green) after 2 h from LPS treatment. Nuclear staining was performed with DRAQ5 (scale bar: 20 µm). B: Cell proliferation assayed via a BrdU kit and expressed as Europium (Eu) counts at 3 h and 24 h ($n = 3$). C: QRT-PCR analyses of *α-SMA* and *COL1* 3 h and 24 h ($n = 3$). Histograms represent the mean ± SD. ***$p < 0.001$ *vs*. NT, Student's *t* test.

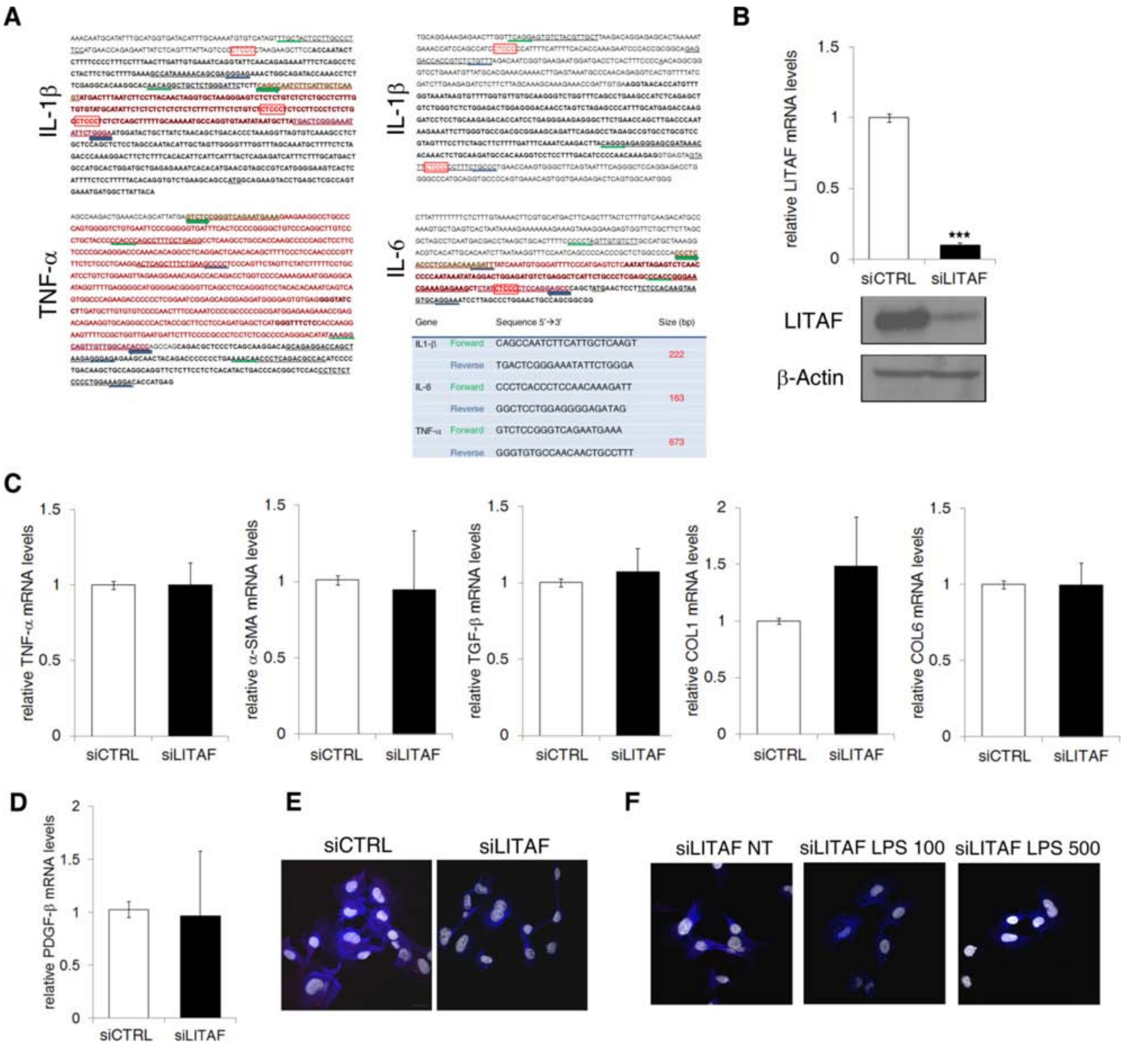

Gene		Sequence 5'→3'	Size (bp)
IL1-β	Forward	CAGCCAATCTTCATTGCTCAAGT	222
	Reverse	TGACTCGGGAAATATTCTGGGA	
IL-6	Forward	CCCTCACCCTCCAACAAAGATT	163
	Reverse	GGCTCCTGGAGGGGAGATAG	
TNF-α	Forward	GTCTCCGGGTCAGAATGAAA	673
	Reverse	GGGTGTGCCAACAACTGCCTTT	

Supplementary Figure S5: LITAF binding and silencing. A. CTCCC repeats (red square) within IL-1β, IL-6 and TNF-α promoters. Forward (green) and reverse (blue) primers are indicated in the promoter sequence. Effective primers for ChIP analyses are listed in the table. **B.** Expression of LITAF mRNA (upper panel) and protein (lower panel) in siCTRL and siLITAF LX-2 cells ($n = 3$). **C.** QRT-PCR analyses of *TNF-α, α-SMA, TGF-β, COL1, COL6* and of (D) *PDGFR-β* ($n = 3$). **D–E.** Representative confocal imaging (scale bar: 20 μm) of α-SMA (blue) and nuclei (DRAQ5, white) in (E) siCTRL and siLITAF LX-2 cells and in (F) siLITAF cells after 2 h treatment with 100 ng/ml and 500 ng/ml LPS ($n = 3$). Histograms represent the mean ± SD. ***$p < 0.001$ for comparison between siCTRL and siLITAF cells, Student's *t* test.

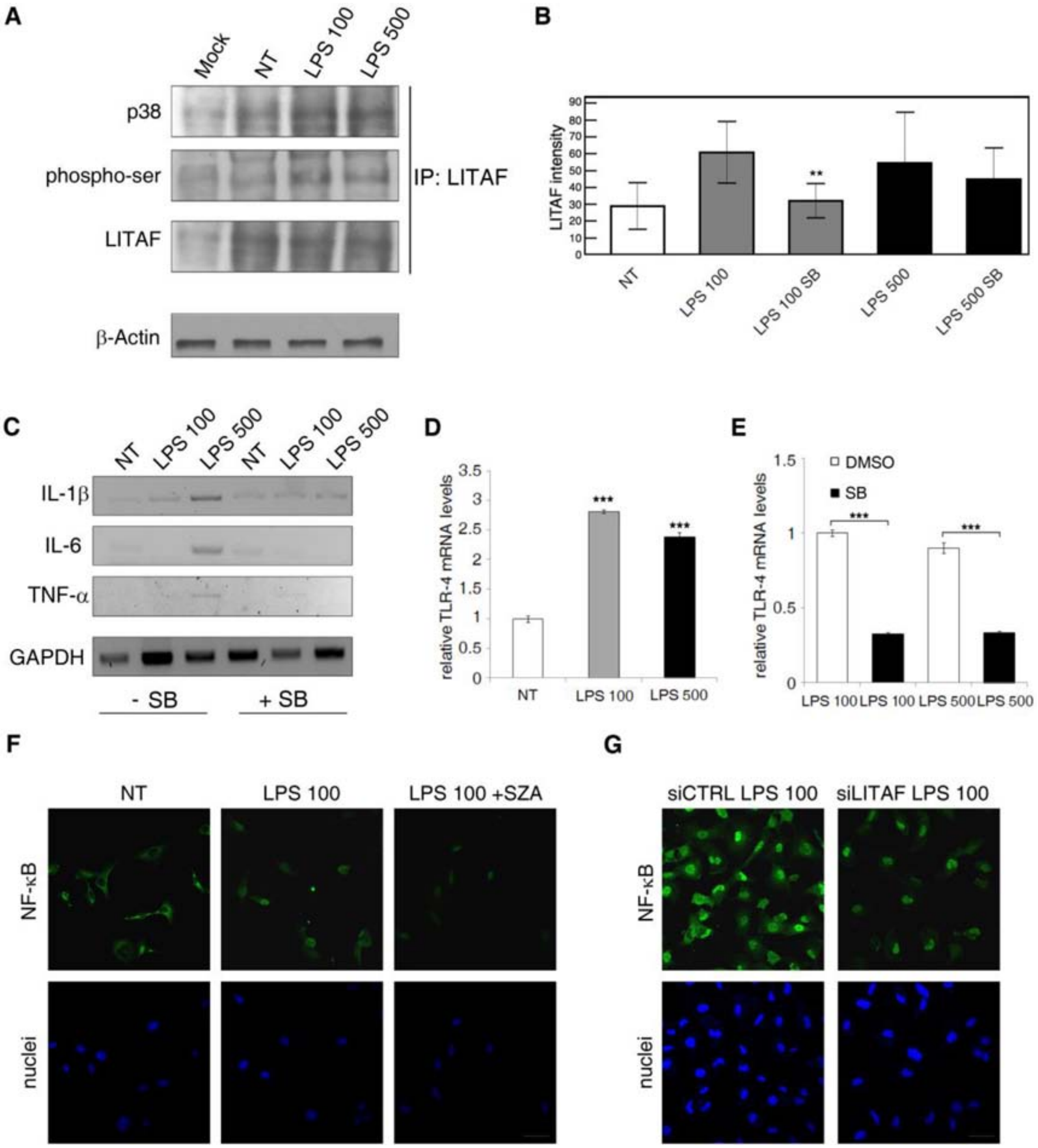

Supplementary Figure S6: p38MAPK interacts with LITAF and its inhibition affects LPS-induced TLR-4 transcription in LX-2 cells. A. Immunoprecipitation was performed with anti-LITAF antibody, or with equivalent amounts of non-specific anti-mouse immunoglobulin (Mock), and revealed with anti-p38MAPK, phospho-serine and LITAF. β-actin was probed as loading control ($n = 2$). **B.** Quantitative imaging analysis of LITAF nuclear translocation after 2 h from LPS addition in SB203850 pre-treated LX-2 cells. Mann-Whitney *U* test. **C.** ChIP analysis of LITAF binding to IL-1β, IL-6 and TNF-α promoters amplified by qPCR. Input DNA was used to assess GAPDH transcript for normalization (data presented are representative of two independent experiments). **D–E.** QRT-PCR analyses of TLR-4 mRNA in LX-2 cells under LPS 100 ng/ml and 500 ng/ml exposure in the absence (D) or presence (E) of SB203850 ($n = 3$). Histograms represent the mean ± SD. ***$p < 0.001$ *vs.* NT or vehicle (DMSO), Student's *t* test. **F–G.** Representative imaging (scale bar: 40 μm) of p65NF-κB (green) and nuclei (blue) after 2 h from different stimuli in LX-2 cells ($n = 2$). Histograms represent the mean ± SD. ***$p < 0.001$ *vs*. NT, Student's *t* test nel pannello B ci sono due asterischi ($p < 0.01$?).

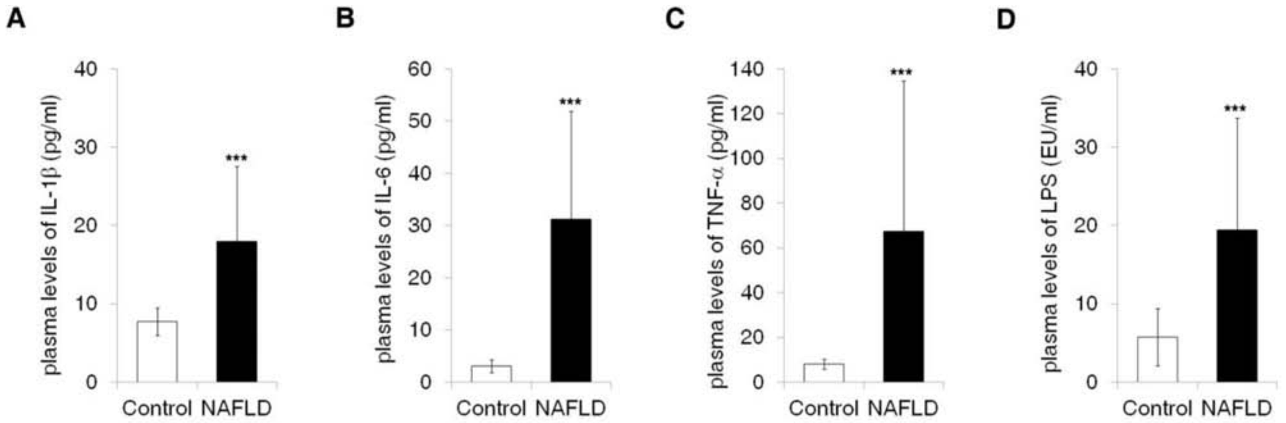

E

	IL-1β (pg/ml)	IL-6 (pg/ml)	TNF-α (ng/ml)	LPS (EU/ml)	LITAF+/ HSCs	STEATOSIS	INFLAMMATION	BALLOONING	FIBROSIS	NASH
IL-1β (pg/ml)				0.625***	0.314*	-0.065	0.466**	0.030	0.115	0.201
IL-6 (pg/ml)	-0.0913		0.547***	-0.181	-0.156	0.225	-0.114	-0.253	0.013	-0.032
TNF-α (ng/ml)	-0.010	0.547***		-0.042	0.246	0.120	0.104	-0.112	0.187	0.236
LPS (EU/ml)	0.625***	-0.181	-0.042		0.242	-0.013	0.512***	0.231	0.353*	0.374*
LITAF+/ HSCs	0.314*	-0.156	0.246	0.242		0.125	0.342*	0.285	0.691***	0.665***
STEATOSIS	-0.065	0.225	0.120	-0.013	0.125		0.032	-0.178	0.295	0.175
INFLAMMATION	0.466**	-0.114	0.104	0.512***	0.342*	0.032		0.365*	0.204	0.496**
BALLOONING	0.030	-0.253	-0.112	0.231	0.285	-0.178	0.365*		0.113	0.535***
FIBROSIS	0.115	0.013	0.187	0.353*	0.691***	0.295	0.204	0.113		0.626***
NASH	0.201	-0.032	0.236	0.374*	0.665***	0.175	0.496**	0.535***	0.626***	

Supplementary Figure S7: IL-1β, IL-6, TNF-α and LPS plasma levels increase in children with NAFLD. **A–D.** Histograms reported mean value ± SD of (A) IL-1β, (B) IL-6, (C) TNF-α and (D) LPS plasma levels in 8 healthy children (Control) and in 40 age-matched subjects with NAFLD. ***$p < 0.001$ for NAFLD *vs*. Control, Student's *t* test. **E.** Values of Pearson's correlations for IL-1β, IL-6, TNF-α and LPS plasma levels and other histological features in paediatric patients with NAFLD ($n = 40$).

Supplementary Table S1: List of primary antibodies used in western blotting and immunostaining

	Antibody Name	Company	Dilution
WB	Rabbit anti-LITAF	Santa Cruz Biotechnology (sc-66944)	1:1000
	Mouse anti-LITAF	Santa Cruz Biotechnology (sc-166719)	1:1000
	Rabbit anti-p38MAPK	Cell Signaling Technology (#9212)	1:1000
	Rabbit anti-phospho-p38MAPK (T180+Y182)	Abcam (ab32557)	1:500
	Rabbit anti-Phosphoserine	Novus Biologicals (NB100–1953)	1:500
	Rabbit anti-p65NFκB	Santa Cruz Biotechnology (sc-109)	1:500
	Rabbit anti-β-actin	Santa Cruz Biotechnology (sc-1616-R)	1:1000
IM	Rabbit anti-LITAF	Santa Cruz Biotechnology (sc-66944)	1:250
	Mouse anti-α-SMA	Novus Biologicals (NB600–536)	1:200
	Mouse anti-CK8/18	Vector Laboratories	1:100
	Mouse anti-CD68	Abcam (ab955)	1:200
	Mouse anti-CD163	Leica Biosystem	1:200

Supplementary Table S2: RT-qPCR primers for quantification of gene expression

Target gene (human)	Primers Code
GAPDH	Hs99999905_m1
LITAF	Hs00191583_m1
ACTA2	Hs00909449_m1
COLVIα1	Hs00242448_m1
COLIα1	Hs00164004_m1
TGF-β1	Hs.PT.49.1806981
TLR-4	Hs01060206_m1
IL-1β	Hs00174097_m1
IL-6	Hs00174131_m1
PDGFR- β	Hs.PT.56a.22892761
**Target gene (mouse)**	**Primers Code**
Gapdh	Mm.PT.39a.1
Litaf	Mm.PT.56a.33549312
Acta2	Mm.PT.56a.30217320
Tgf-β1	Mm.PT.56a.43479940
Col1α1	Mm.PT.56a.17374081
Col6α1	Mm.PT.56a.10027971
Tlr-4	Mm.PT.56a.41643680
